# Supplementary material for: A genome-wide association study of serum proteins reveals shared loci with common diseases
Source: Nat Commun. 2022 Jan 25;13:480. doi: 10.1038/s41467-021-27850-z (PMC8789779; doi:10.1038/s41467-021-27850-z)
Supplement: Supplementary file 1 — Supplementary Information [file 41467_2021_27850_MOESM1_ESM.pdf]

# Supplementary Information

## A genome-wide association study of serum proteins reveals shared loci with common diseases

Alexander Gudjonsson[1,*], Valborg Gudmundsdottir[1,2,*], Gisli T Axelsson[1,2], Elias F Gudmundsson[1], Brynjolfur G Jonsson[1], Lenore J Launer[3], John R Lamb[4], Lori L Jennings[5], Thor Aspelund[1,2], Valur Emilsson[1,2,**] & Vilmundur Gudnason[1,2,**]

[1]Icelandic Heart Association, Holtasmari 1, 201 Kopavogur, Iceland.
[2]Faculty of Medicine, University of Iceland, 101 Reykjavik, Iceland.
[3]Laboratory of Epidemiology and Population Sciences, Intramural Research Program, National Institute on Aging, Bethesda, MD 20892-9205, USA.
[4]GNF Novartis, 10675 John Jay Hopkins Drive, San Diego, CA 92121, USA.
[5]Novartis Institutes for Biomedical Research, 22 Windsor Street, Cambridge, MA 02139, USA.

[*]These authors contributed equally
[**]These authors jointly supervised this work

Correspondence: v.gudnason@hjarta.is

**Supplementary Material** ........ **1**
Supplementary Note 1: Novelty estimates for pQTLs reported in the current study ........ 3
Supplementary Note 2: Replication of pQTLs between INTERVAL and AGES cohorts ........ 5
Supplementary Note 3: GWAS of the serum protein co-regulatory network ........ 7
Supplementary Figures ........ 12
Supplementary References ........ 35

## Supplementary Note 1: Novelty estimates for pQTLs reported in the current study

The current study is a genome-wide analysis of ~7.5M variants imputed using the HRC reference panel in 5,368 AGES participants and measurements of 4,782 SOMAmers. We compared the pQTLs identified in the current study to previously reported studies: a) the INTERVAL-study GWAS[1] of 3,283 SOMAmers in 3,301 individuals based on 10.6 million imputed (combined 1000 Genomes Phase 3-UK10K reference panel) autosomal variants, b) our previously published[2] *cis* pQTL (300kb window up- and downstream of gene boundaries) and *cis*-to-*trans* pQTLs analysis for 4,783 SOMAmers in 3,200 AGES participants using genetic variants imputed using the 1000 Genomes v3 reference panel and c) a recent Illumina exome array analysis[3] of 54,469 variants and 4,782 SOMAmers in up to 5,343 AGES participants, d) 17 additional proteogenomic studies with a more limited protein coverage (Supplementary Data 5).

In the current study, genetic signals were defined as shared across SOMAmers if the lead variants were in strong LD ($r^2$>0.9). Therefore, the same LD threshold was used to define shared genetic signals across studies based on reported lead variants. However, the study comparison was also performed using a more stringent threshold ($r^2$>0.5). In addition, we performed a lookup of lead independent variants in the current study in the publicly available summary statistics from the INTERVAL study[1].

As shown in Supplementary Table 1 and Supplementary Fig. 4, 1,452 (36%) associations were considered novel at the primary LD threshold and 1,188 (29%) remained so at the more stringent LD threshold. The majority of proteins (1,922 or 92%) for which we find a pQTL have a previously reported pQTL, with a large proportion originating from our recent exome chip study[3], while the current study increases considerably the number of known genetic signals for serum protein levels (38% of genetic signals defined as novel at the primary LD threshold).

Finally, we considered novelty at the locus level, in addition to an evaluation based on the independent signals. Combining independent signals within 300kb of each other, we identified 772 loci associated with protein levels. Of those, 94 (12%) are novel, in the sense that they are >500kb away from lead pQTL variants reported in other studies. Of 3,079 locus-protein associations, 565 (18%) are previously unreported. If we exclude our own exome array analysis in the AGES cohort[3], we find that 17% of the loci are novel and 40% of the locus-protein associations.

**Supplementary Table 1** – Comparison of independent pQTLs (association, genetic signal and protein) identified in the current study compared to previously reported proteogenomic studies. The comparison is shown at two different LD thresholds.

| | | | | Known | | Addition | | Novel | |
|---|---|---|---|---|---|---|---|---|---|
| **LD threshold (r2)** | **Study compared to** | **Type** | **Total** | **count** | **%** | **count** | **%** | **count** | **%** |
| 0.9 | Sun et al. 2018 | assoc | 4035 | 1304 | 32.3 | NA | NA | 2731 | 67.7 |
| 0.9 | Sun et al. 2018 | signal | 2024 | 707 | 34.9 | 188 | 9.3 | 1129 | 55.8 |
| 0.9 | Sun et al. 2018 | protein | 2091 | 387 | 18.5 | 686 | 32.8 | 1018 | 48.7 |
| 0.9 | Emilsson et al. 2018 | assoc | 4035 | 1087 | 26.9 | NA | NA | 2948 | 73.1 |
| 0.9 | Emilsson et al. 2018 | signal | 2024 | 428 | 21.1 | 55 | 2.7 | 1541 | 76.1 |
| 0.9 | Emilsson et al. 2018 | protein | 2091 | 485 | 23.2 | 766 | 36.6 | 840 | 40.2 |
| 0.9 | Emilsson et al. 2020 | assoc | 4035 | 1572 | 39.0 | NA | NA | 2463 | 61.0 |
| 0.9 | Emilsson et al. 2020 | signal | 2024 | 355 | 17.5 | 30 | 1.5 | 1639 | 81.0 |
| 0.9 | Emilsson et al. 2020 | protein | 2091 | 728 | 34.8 | 998 | 47.7 | 365 | 17.5 |
| 0.9 | Other | assoc | 4035 | 332 | 8.2 | NA | NA | 3703 | 91.8 |
| 0.9 | Other | signal | 2024 | 202 | 10.0 | 231 | 11.4 | 1591 | 78.6 |
| 0.9 | Other | protein | 2091 | 73 | 3.5 | 394 | 18.8 | 1624 | 77.7 |
| 0.9 | Any | assoc | 4035 | 2583 | 64.0 | NA | NA | 1452 | 36.0 |
| 0.9 | Any | signal | 2024 | 1060 | 52.4 | 204 | 10.1 | 760 | 37.5 |
| 0.9 | Any | protein | 2091 | 1015 | 48.5 | 907 | 43.4 | 169 | 8.1 |
| 0.5 | Sun et al. 2018 | assoc | 4035 | 1345 | 33.3 | NA | NA | 2690 | 66.7 |
| 0.5 | Sun et al. 2018 | signal | 2024 | 734 | 36.3 | 244 | 12.1 | 1046 | 51.7 |
| 0.5 | Sun et al. 2018 | protein | 2091 | 405 | 19.4 | 668 | 31.9 | 1018 | 48.7 |
| 0.5 | Emilsson et al. 2018 | assoc | 4035 | 1252 | 31.0 | NA | NA | 2783 | 69.0 |
| 0.5 | Emilsson et al. 2018 | signal | 2024 | 522 | 25.8 | 92 | 4.5 | 1410 | 69.7 |
| 0.5 | Emilsson et al. 2018 | protein | 2091 | 531 | 25.4 | 720 | 34.4 | 840 | 40.2 |
| 0.5 | Emilsson et al. 2020 | assoc | 4035 | 2009 | 49.8 | NA | NA | 2026 | 50.2 |
| 0.5 | Emilsson et al. 2020 | signal | 2024 | 636 | 31.4 | 57 | 2.8 | 1331 | 65.8 |
| 0.5 | Emilsson et al. 2020 | protein | 2091 | 919 | 44.0 | 807 | 38.6 | 365 | 17.5 |
| 0.5 | Other | assoc | 4035 | 435 | 10.8 | NA | NA | 3600 | 89.2 |
| 0.5 | Other | signal | 2024 | 266 | 13.1 | 296 | 14.6 | 1462 | 72.2 |
| 0.5 | Other | protein | 2091 | 104 | 5.0 | 363 | 17.4 | 1624 | 77.7 |
| 0.5 | Any | assoc | 4035 | 2847 | 70.6 | NA | NA | 1188 | 29.4 |
| 0.5 | Any | signal | 2024 | 1233 | 60.9 | 197 | 9.7 | 594 | 29.3 |
| 0.5 | Any | protein | 2091 | 1160 | 55.5 | 762 | 36.4 | 169 | 8.1 |

## Supplementary Note 2: Replication of pQTLs between INTERVAL and AGES cohorts

*Replication of INTERVAL study pQTLs in AGES*

We performed a lookup of previously reported pQTLs from the INTERVAL study[1] (n = 3,301). The comparison was based on the reported independent variants from a conditional analysis, however comparing univariate effect sizes and P-values to our GWAS results for consistency.

Replication of pQTLs from the INTERVAL study was good, with 84.6% of reported associations directionally consistent in AGES and 75.6% both directionally consistent and nominally significant (Supplementary Table 2, Supplementary Fig. 5). While the proportion of study-wide significant associations in AGES increased with a stronger reported significance in the INTERVAL study as expected, rather surprisingly the proportion of non-significant associations remained constant around 20% independent of the reported significance (Supplementary Fig. 5C). This was explained by a large proportion of pQTLs reported in the INTERVAL study that are located on chromosome 19, where a strikingly high proportion was not replicated in AGES (Supplementary Fig. 6A). A further investigation narrowed this region down to the *NLRP12* locus (chr 19, 54,319,624-54,327,869), a *trans* hotspot reported in the INTERVAL study with a total of 391 associations, whereof only 20 were nominally significant in the current study (Supplementary Fig. 4B), with the minimum observed P-value = 0.002 in AGES at this locus compared to a minimum P-value = $6.5\times10^{-244}$ in the INTERVAL study. Given the strong observed significance in the INTERVAL study, the associations at this locus are unlikely to be false positives, however the discrepancies between the two studies at this locus may rather reflect some cohort-specific attributes, as for instance the INTERVAL study is based on plasma samples compared to serum samples in AGES and there is a large age gap between the two cohorts. More specifically, the INTERVAL cohort consists of young (mean age ~44 years), healthy blood donors of European descent and recruited in England and the proteins are measured in plasma samples, while the population-based AGES cohort consists of elderly (mean age ~76 years) Icelanders, many of whom suffer from chronic diseases and the proteins are measured in serum samples. Others have suggested that differences in observed pQTLs in this locus may be driven by sample handling and white blood cell lysis[4]. The lead variant in the INTERVAL study at this locus (rs62143197) is an intron variant in *NLRP12*, which is also an eQTL for *NLRP12* in GTEx (P = $3.2\times10^{-15}$ for whole blood, https://gtexportal.org/home/). A lookup of this variant in UKBB associations[5] (http://geneatlas.roslin.ed.ac.uk/) revealed strong

associations with monocyte percentage (beta = -0.12, $P = 3.4\times10^{-155}$) and counts (beta = -0.009, $P = 1.5\times10^{-141}$).

When the *NLRP12* locus was excluded from the pQTL comparison, 97.6% of associations reported in the INTERVAL study were directionally consistent in the current study and 93.3% were both directionally consistent and nominally significant (Supplementary Table 2, Supplementary Fig. 5).

*Replication of AGES pQTLs in the INTERVAL study*

We next performed a lookup of the pQTLs identified in the current study (n = 5,368) in summary statistics from the INTERVAL study[1] (n = 3,301). The comparison was based on the lead independent variants from the conditional analysis, however comparing univariate effect sizes and P-values for consistency between the two studies and restricted to the 4,028 associations that were study-wide significant in both the joint model (from the conditional analysis) and the univariate GWAS in AGES.

Of the 2,690 associations that could be compared between the studies, we found 94.2% to be directionally consistent and 82% were both directionally consistent and at least nominally significant ($P < 0.05$) in the INTERVAL study (Supplementary Table 2). When we restricted the comparison to 645 pQTLs defined as novel in the current study (see Supplementary Note 1) and with information available in the INTERVAL study, we still found 90% to be directionally consistent and 64% to be both directionally consistent and nominally significant ($P < 0.05$).

**Supplementary Table 2** – Overview of reported independent pQTLs (linear regression) in the INTERVAL study[1] with their replication status in AGES and vice versa. The INTERVAL study results are also shown excluding the *NLRP12* locus, a *trans* hotspot in the INTERVAL study. For the current AGES study, we show the results for all associations and only those defined as novel.

| **Discovery** | **Sun et al. 2018** | **Sun et al. 2018 (excluding the NLRP12 locus)** | **AGES** | **AGES (novel only)** |
|---|---|---|---|---|
| **Replication** | **AGES** | **AGES** | **Sun et al. 2018** | **Sun et al. 2018** |
| Associations (n) | 1905 | 1514 | 2690 | 645 |
| SNPs (n) | 1046 | 1037 | 1558 | 467 |
| SOMAmers (n) | 1359 | 1022 | 1605 | 534 |
| Directionally consistent (n (%)) | 1611 (84.6) | 1476 (97.5) | 2533 (94.2) | 581 (90.1) |
| Nominally significant (P<0.05) in replication (n (%)) | 1441 (75.6) | 1421 (93.9) | 2219 (82.5) | 414 (64.2) |
| Directionally consistent and nominally significant (P<0.05) in replication (n (%)) | 1417 (74.4) | 1413 (93.3) | 2198 (81.7) | 412 (63.9) |

## Supplementary Note 3: GWAS of the serum protein co-regulatory network

We regressed 54 Eigenproteins (1st and 2nd PCs) on 7.5 million variants that were assayed or imputed in all 5,368 AGES participants with protein data. Combined, the 1st and 2nd PCs capture on average 45% of the variance in each serum protein module. We assumed an additive linear model for each module’s Eigenprotein. Applying the conventional P-threshold of $5.0\times10^{-8}$ for genome-wide significance when a linkage disequilibrium (LD) $r^2$<0.8 is used for independent variants, we find that 24 (89%) out of 27 modules are associated with at least one independent network-associated protein SNP (npSNP) (Supplementary Table 3). This is a marked increase (26%) in number of identified npSNPs compared to previous results based on 1.5 million markers in 3,219 AGES participants[2]. Applying a naive conservative Bonferroni correction for number of variants and Eigenproteins tested, 18 (67%) modules showed significant association to at least one independent npSNP at $P<1.2\times10^{-10}$. Often, certain npSNPs are associated with more than one protein module that is consistent with their relationship[2].

When the significant npSNPs listed in Supplementary Table 3 were tested against all proteins we considered associations at $P < 1\times10^{-7}$ significant, using Bonferroni correction for number of npSNPs and proteins tested. The majority of npSNPs were associated with many individual proteins in *cis* and/or *trans* (mostly *trans*), while the proteins that were affected comprised the module(s) associated with the corresponding npSNP which is in line with previous

observations[2]. For instance, four npSNPs across three chromosomal sites including a region harboring *APOE/TOMM40*, were associated with either 1st or 2nd PCs for the protein module 11 (PM11) (Supplementary Table 3). These variants were associated with 136 proteins in *cis* and/or *trans* including 96% of all proteins that constitute PM11. These results show that npSNP are linked to many co-regulated proteins and are underlying the architecture of the serum protein network.

The npSNPs associated with the module PM11, are also known variants in genome-wide association studies (GWAS) linked to Late-Onset Alzheimer´s Disease (LOAD), lipoproteins and coronary heart disease (CHD) (Supplementary Table 3). In fact, the majority of the npSNPs listed in Supplementary Table 3 have previously been linked to an array of clinical traits and complex diseases. For instance, a recent GWAS study identified a number of variants associated with immunoglobulin G N-glycosylation (IgGG)[6], but aberrant glycosylation of IgG has been linked to a number of age-related disease outcomes[7]. Here we find that the npSNPs rs35590487 and rs35592422 associated with the protein module PM20 (Supplementary Table 3), are also found to be associated with IgGG at chromosome 14[6,8]. More to the point, rs35590487 and rs35592422 were associated with 37 proteins including 56% of all proteins that comprise PM20. Other striking examples include the npSNPs associated with the 1st or 2nd PCs of the protein modules PM1, PM7, PM12-PM15 (Supplementary Table 3), but these are known GWAS risk variants for age-related macular degeneration (AMD)[9]. These and other examples[2], highlight the genetic architecture of the serum protein network and demonstrate that the protein network and the genetic risk of diseases is intimately connected.

**Supplementary Table 3** – Genetic variants associated with module $E^{(q)}$s. Identification of genetic variants associated (linear regression) with different modules $E^{(q)}$s (q is the annotation for a specific module). Associations at the conventional genome-wide significant $P<5.0\times10^{-8}$ or naïve Bonferroni corrected $P<1.2\times10^{-10}$ are reported. N/A, not applicable. GWAS, phenotypes associated with the corresponding npSNP at $P<5.0\times10^{-8}$ as reported in the PhenoScanner and/or the GWAS catalogue.

| Module | Cluster | 1st $E^{(q)}$ npSNP | 2nd $E^{(q)}$ npSNP | npSNP chr. | P-value npSNP | N proteins affected | Known GWAS** | *Cis* expression QTL (tissue)* |
|---|---|---|---|---|---|---|---|---|
| **PM1** | I | rs537179722 | | 6 | 6.9E-12 | 36 | RA, AMD, LC | SKIV2L (many) |
| | | | rs887829 | 2 | 7.9E-43 | 14 | Bilirubin | UGT1A3 (liver) |
| **PM2** | II | rs704 | | 17 | 2.4E-10 | 846 | OPG | TMEM199 (many) |
| | | rs12439785 | | 15 | 3.2E-09 | 7 | Myopia | None |
| | | | rs704 | 17 | 1.8E-12 | 846 | OPG | TMEM199 (many) |
| **PM3** | II | rs11574452 | | 4 | 4.5E-16 | 289 | Albumin | PF4 (fibroblasts) |
| | | | rs115008613 | 1 | 9.9E-09 | None | None | None |
| | | | rs73225360 | X | 4.2E-08 | None | None | None |
| **PM4** | II | rs11574452 | | 4 | 6.7E-10 | 289 | Albumin | PF4 (fibroblasts) |
| | | | rs10761741 | 10 | 4.2E-08 | 19 | MPV, TG | NRBF2 (fibroblasts) |
| | | | rs704 | 17 | 1.6E-37 | 846 | OPG | TMEM199 (many) |
| **PM5** | II | rs2731674 | | 5 | 1.5E-09 | 115 | Height | F12 (liver) |
| | | | rs2345872 | 2 | 4.2E-08 | 8 | None | FMNL2 (fibroblasts) |
| | | | rs75931041 | 11 | 2.0E-08 | None | None | None |
| **PM6** | II | rs73088258 | | 7 | 3.8E-08 | 6 | None | None |
| **PM7** | II | rs241779 | | 17 | 1E-302 | 729 | AMD | TMEM199 (many) |
| | | | rs241775 | 17 | 1E-302 | 728 | AMD | TMEM199 (many) |
| **PM8** | II | | rs704 | 17 | 2.6E-19 | 846 | OPG | TMEM199 (many) |
| **PM9** | II | rs1178713 | | 3 | 4.1E-08 | 14 | None | None |
| **PM10** | II | rs704 | | 17 | 3.1E-72 | 846 | OPG | TMEM199 (many) |
| | | | rs704 | 17 | 7.9E-143 | 846 | OPG | TMEM199 (many) |
| | | | rs11574452 | 4 | 1.9E-11 | 289 | Albumin | PF4 (fibroblasts) |
| | | | rs597808 | 12 | 1.1E-08 | 15 | CRC, HT-H, SLE | ALDH2 (esophagus) |
| **PM11** | III | rs483082 | | 19 | 4.8E-160 | 59 | LOAD, TG | APOE (skin) |
| | | rs1355537 | | 3 | 3.1E-15 | 41 | None | BCHE (nerve) |
| | | | rs429358 | 19 | 3.0E-231 | 63 | LOAD, LDL, CHD | TOMM40 (brain) |
| | | | rs1260326 | 2 | 1.4E-09 | 26 | TG, CRP, CHOL | NRBP1 (many) |
| **PM12** | III | | rs704 | 17 | 1.9E-16 | 846 | OPG | TMEM199 (many) |
| | | | rs3117116 | 6 | 9.9E-12 | 25 | RA, MS | HLA-DRB5 (many) |
| | | | rs528298 | 1 | 5.9-09 | 198 | AMD | CFHR1/3 (many) |
| **PM13** | III | rs528298 | | 1 | 2.2E-14 | 198 | AMD | CFHR1/3 (many) |
| | | rs1042663 | | 6 | 4.5E-13 | 152 | RA, AMD, NV, MPV | SKIV2L (many) |
| | | rs171360 | | 17 | 7.8E-10 | 526 | None | TMEM97 (thyroid) |
| | | rs74480769 | | 5 | 3.6E-08 | 111 | None | C7 (many) |
| | | | rs704 | 17 | 2.2E-24 | 846 | OPG | TMEM199 (many) |
| | | | rs74480769 | 5 | 1.8E-10 | 111 | None | C7 (many) |
| | | | rs528298 | 1 | 5.1E-10 | 198 | AMD | CFHR1/3 (many) |

| | | | | | | | | |
|---|---|---|---|---|---|---|---|---|
| | | | rs115009784 | 6 | 7.9E-10 | 53 | AMD, RA | DXO (many) |
| | | | rs11574452 | 4 | 2.9E-09 | 289 | Albumin | PF4 (fibroblasts) |
| **PM14** | III | rs74480769 | | 5 | 4.1E-11 | 111 | None | C7 (many) |
| | | rs1042663 | | 6 | 1.2E-10 | 152 | RA, AMD, NV, MPV | SKIV2L (many) |
| | | rs528298 | | 1 | 1.1E-09 | 198 | AMD | CFHR1/3 (many) |
| | | rs12067507 | | 1 | 2.7E-08 | 56 | None | None |
| **PM15** | III | rs1042663 | | 6 | 2.3E-17 | 152 | RA, AMD, NV, MPV | SKIV2L (many) |
| | | rs528298 | | 1 | 2.7E-17 | 198 | AMD | CFHR1/3 (many) |
| | | rs74480769 | | 5 | 2.4E-14 | 111 | None | C7 (many) |
| | | rs171360 | | 17 | 1.1E-08 | 526 | None | TMEM97 (thyroid) |
| | | | rs115008613 | 1 | 3.7E-08 | None | None | None |
| **PM16** | IV | | rs11574452 | 4 | 1.9E-09 | 289 | Albumin | PF4 (fibroblasts) |
| | | | rs61759306 | 1 | 3.9E-08 | None | RBC, MCHC | SMIM1 (blood) |
| **PM17** | IV | None | None | N/A | N/A | N/A | N/A | N/A |
| **PM18** | IV | rs35120348 | | 12 | 3.7E-09 | 5 | None | METTL21B (blood) |
| **PM19** | IV | None | None | N/A | N/A | N/A | N/A | N/A |
| **PM20** | V | rs35590487 | | 14 | 6.7E-18 | 35 | IgGG | TMEM121 (many) |
| | | rs111648045 | | 2 | 3.7E-08 | 6 (complex) | None | None |
| | | | rs35592422 | 14 | 1.7E-25 | 36 | IgGG | TMEM121 (many) |
| | | | rs2855812 | 6 | 6.0E-12 | 10 | T1D, Asthma, RA | MICB (many) |
| | | | rs55800253 | 11 | 2.8E-08 | None | None | None |
| **PM21** | V | | rs9268833 | 6 | 4.0E-09 | 13 | RA, Asthma | HLA (many) |
| **PM22** | V | | rs3756074 | 4 | 9.2E-09 | 285 | Albumin | PF4 (fibroblasts) |
| **PM23** | V | rs77869924 | | 16 | 2.8E-08 | 3 | None | None |
| | | | rs77625270 | 19 | 3.4E-23 | 1 | None | TIMM44 (blood) |
| | | | rs9411378 | 9 | 4.5E-09 | 65 | LDL, MI, VTE | ABO (many) |
| | | | rs16850360 | 4 | 4.0E-08 | 244 | CAC | PF4 (fibroblasts) |
| **PM24** | V | | rs1250258 | 2 | 1.1E-57 | 7 | CHD, Endometriosis | ATIC (blood) |
| | | | rs5985 | 6 | 5.7E-11 | 3 | ESC | None |
| | | | rs353634 | 11 | 7.8E-09 | 1 | Hair colour | CD44 (artery) |
| **PM25** | V | rs185289117 | | 14 | 3.0E-85 | 42 | None | None |
| | | rs150787591 | | 2 | 7.4E-09 | None | None | None |
| | | rs4016738 | | 3 | 3.2E-08 | 2 | MC | WDR48 (blood) |
| | | | rs80324969 | 14 | 2.5E-25 | 38 | None | CRIP2 (heart) |
| | | | rs3134998 | 6 | 2.7E-09 | 11 | RA, Asthma | HLA (many) |
| **PM26** | V | rs76833488 | | 15 | 5.7E-09 | 15 | None | None |
| | | | rs11574452 | 4 | 1.7E-13 | 289 | Albumin | PF4 (fibroblasts) |
| | | | rs4131289 | 5 | 2.1E-09 | 1 | APTT, CKD | RGS14 (many) |
| **PM27** | V | None | None | | N/A | | N/A | N/A |

*An example of *cis* expression QTL from the GTEx database.

**For a qualified proxy the correlation between npSNP and corresponding lead GWAS SNP was $r^2 \geq 0.8$. Abbreviations: RA, rheumatoid arthritis; AMD, age-related macular degeneration; LC, lymphocyte count; OPG, Osteoprotegerin levels; MPV, mean platelet volume; TG, triglyceride; CRC, colorectal cancer; HT-H, hypothyroidism; SLE, systemic lupus erythrematosus; LOAD, late-onset Alzheimer´s disease; CRP, C-reactive protein;

CHOL, total cholesterol; MS, multiple sclerosis; WM, brain white matter; RBC, red blood count; MCHC, mean corpuscular hemoglobin concentration; IgGG, IgG glycosylation; T1D, type 1 diabetes; VTE, venous thromboembolism; CAC, coronary artery calcium; CHD, coronary heart disease; ESC, end-stage coagulation; MC, monocyte count; APTT, activated partial thromboplastin time ; CKD, chronic kidney disea

## Supplementary Figures

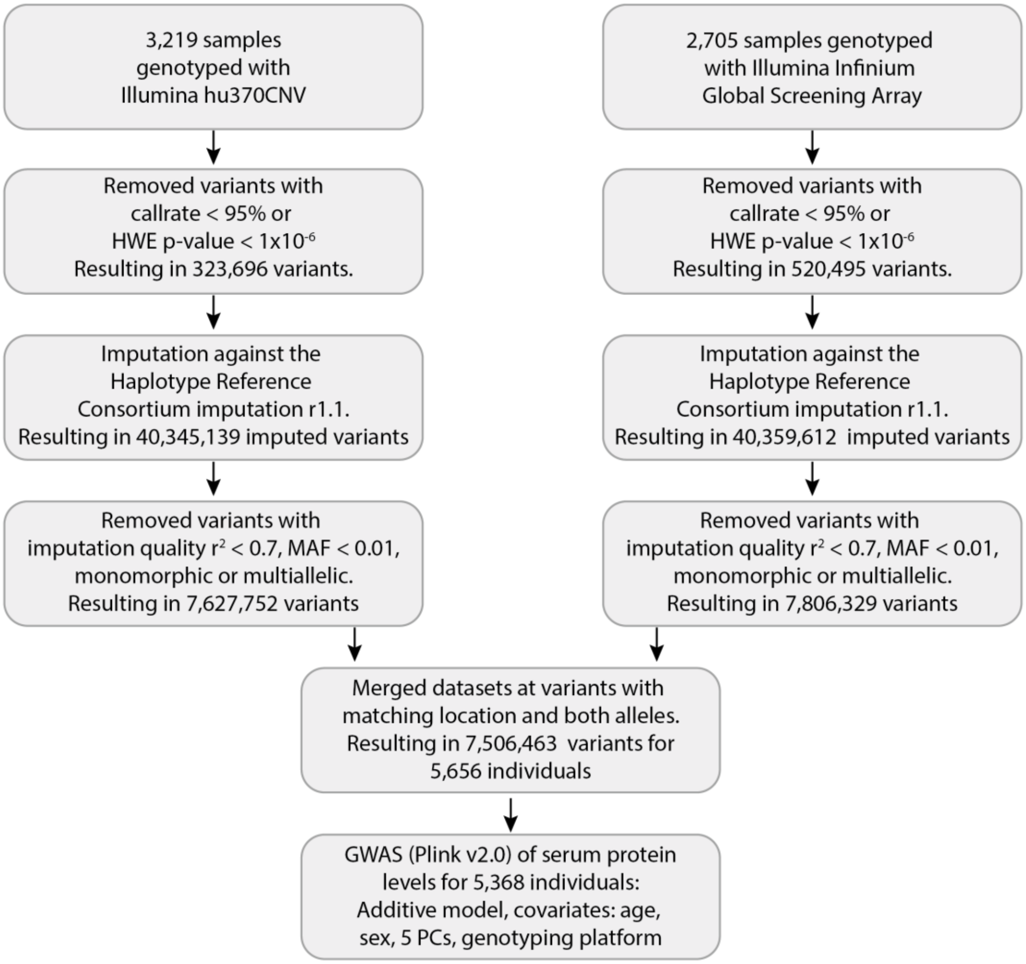


**Supplementary Fig. 1** A flowchart illustrating the preprocessing steps of AGES-Reykjavik genotype data undertaken before the GWAS of serum protein levels. In short, the AGES-Reykjavik samples were genotyped using two different platforms and the resulting genotypes filtered in a similar manner before HRC imputation. The two datasets were merged after post-imputation filtering and used for the GWAS analysis, while including genotype platform among the covariates.

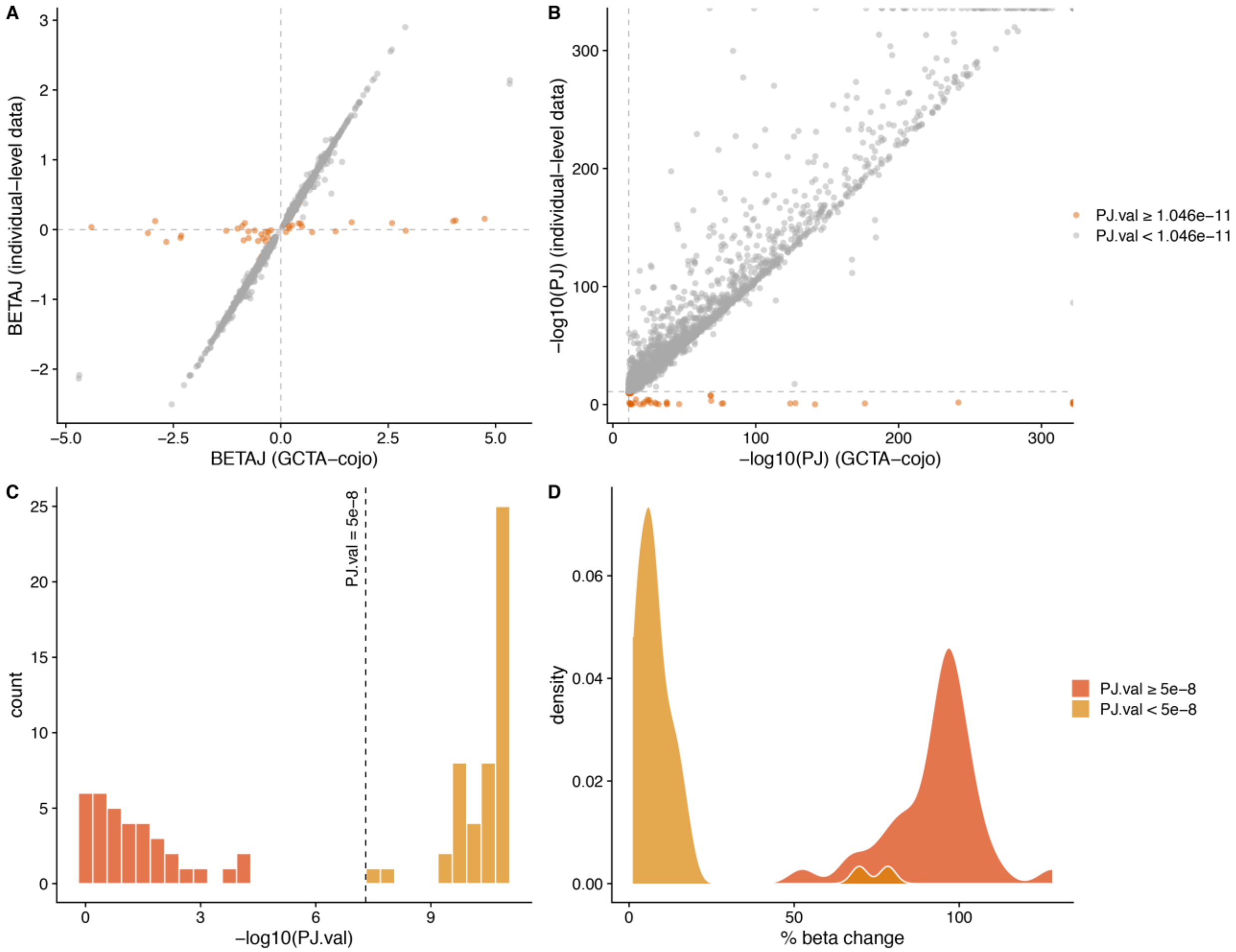


**Supplementary Fig. 2** Comparison of (**A**) beta values and (**B**) –log10(P-values) for joint models of independent SNP-SOMAmer associations obtained by a conditional and joint analysis with GCTA-COJO (x-axis) and a validation analysis (linear regression) using individual level data in AGES (y-axis), colored by study-wide significance in the validation analysis. Of 4,374 SNP-SOMAmer associations, 84 (2%) did not reach study-wide significance in the validation analysis and were thus excluded from the final results. (**C**) The distribution of –log10(P-values) for the 84 SNP-SOMAmer associations not reaching study-wide significance in the validation analysis, further split those into those that reach genome-wide significance or not. (**D**) The distribution of %beta change for the 84 non-validated SNP-SOMAmer associations, stratified by reaching genome-wide significance in the validation analysis or not. The bimodal distribution suggests that a part of the non-validated associations remain borderline significant with a similar effect size (yellow) while the others are attenuated (orange) and may represent artifactual results from the GCTA-COJO analysis.

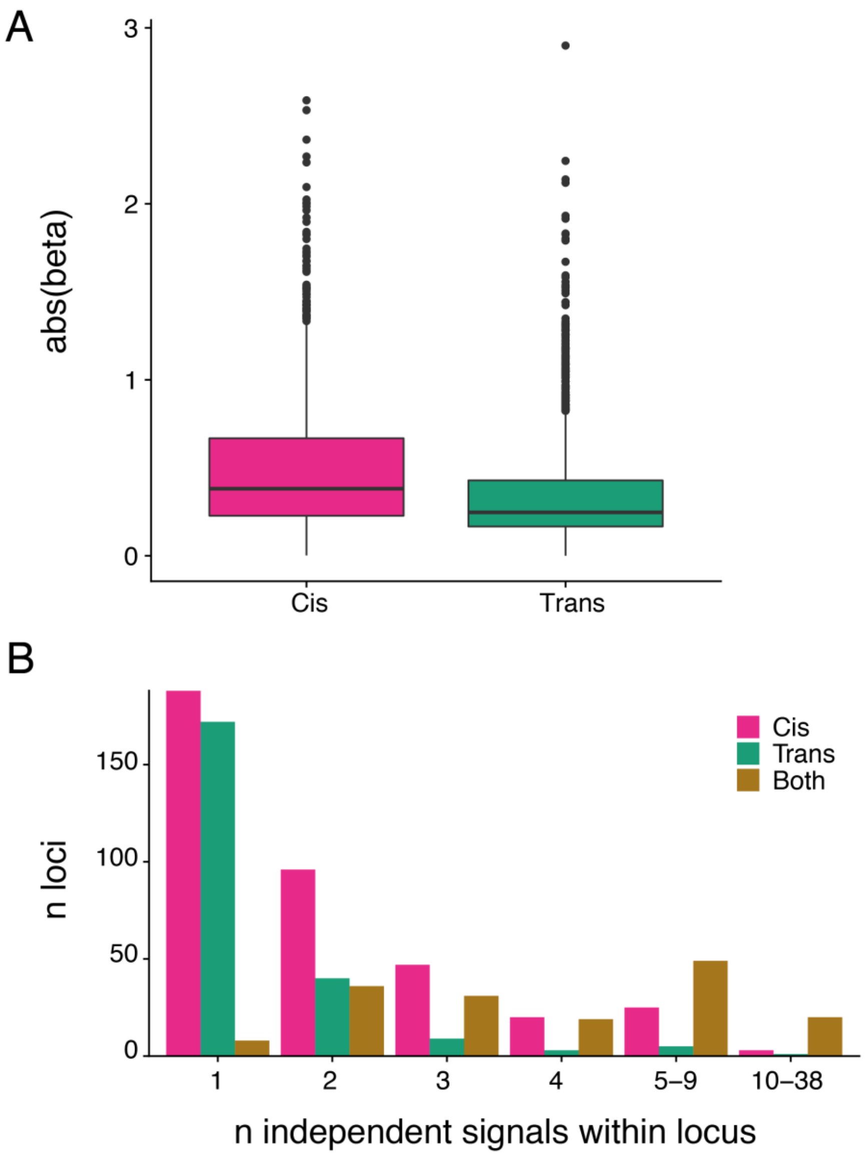


**Supplementary Fig. 3** (**A**) Boxplot comparing effect sizes (absolute beta value) between *cis* (n = 1,415) and *trans* (n = 2,620) associations. Only independent associations (Supplementary Data 3) are included in the comparison and the strongest association (lowest P-value) chosen per protein if targeted by more than one SOMAmer. The median values are 0.38 (*cis*) and 0.25 (*trans*) and are significantly different (two-sided Wilcoxon's P = $1.5\times10^{-6}$). Boxplots indicate median value, 25th and 75th percentile, whiskers extend to smallest/largest value no further than 1.5×IQR, outliers are shown. (**B**) Barplot showing the distribution of study-wide independent signals per locus. Independent signals within 300 kb from each other were considered to share the same locus (see Methods for details).

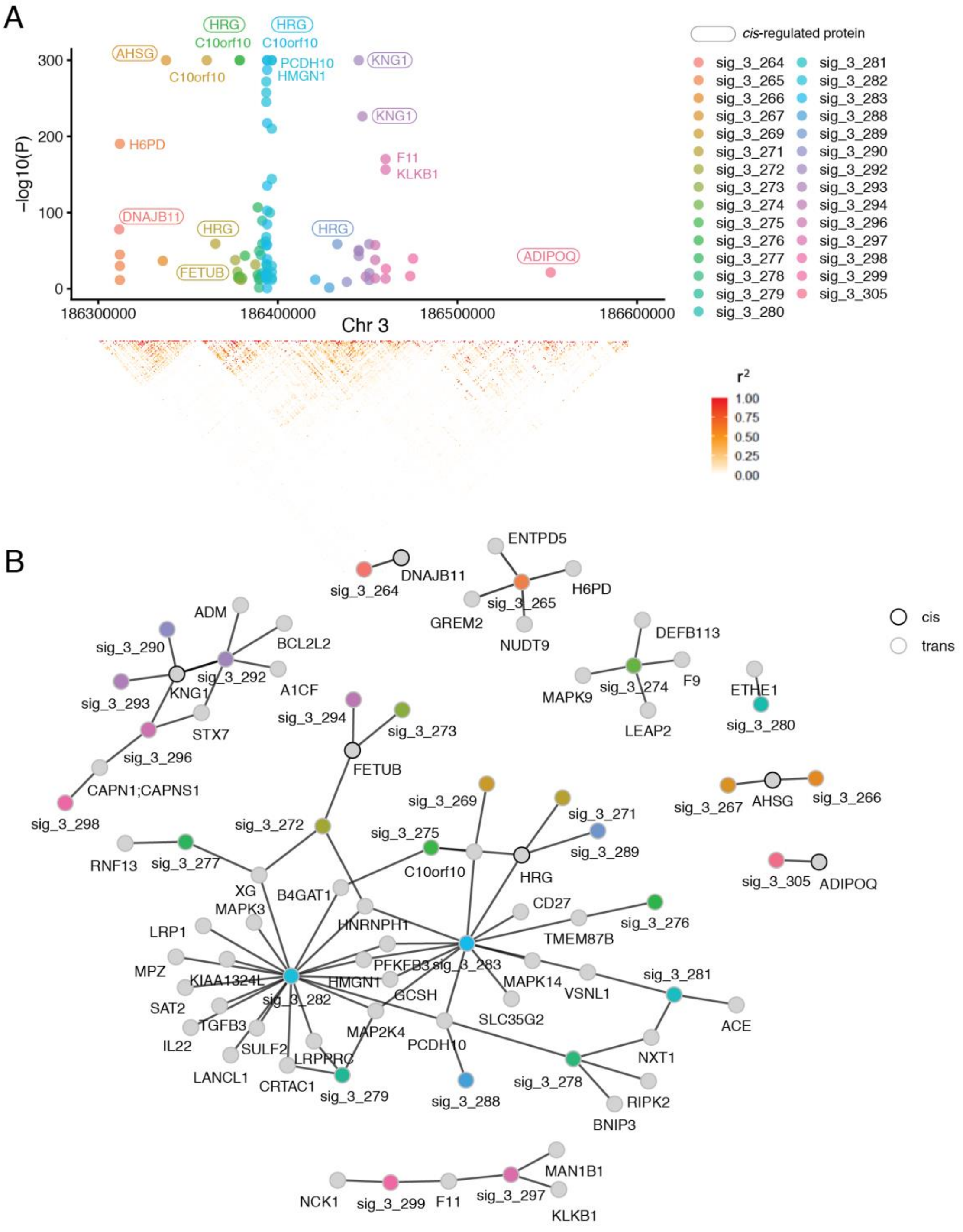


**Supplementary Fig. 4** An overview of a region on chromosome 3 (186.3-186.6 Mb) where 29 independent study-wide significant signals were identified (conditional and joint analysis GCTA-COJO, n = 5,368). (**A**) Each point represents the -log10(P-value) for the associations between a given independent signal and a serum protein. The LD structure in the region (based on AGES data) is shown below. (**B**) The network demonstrates the links between independent signals in the region and serum proteins.

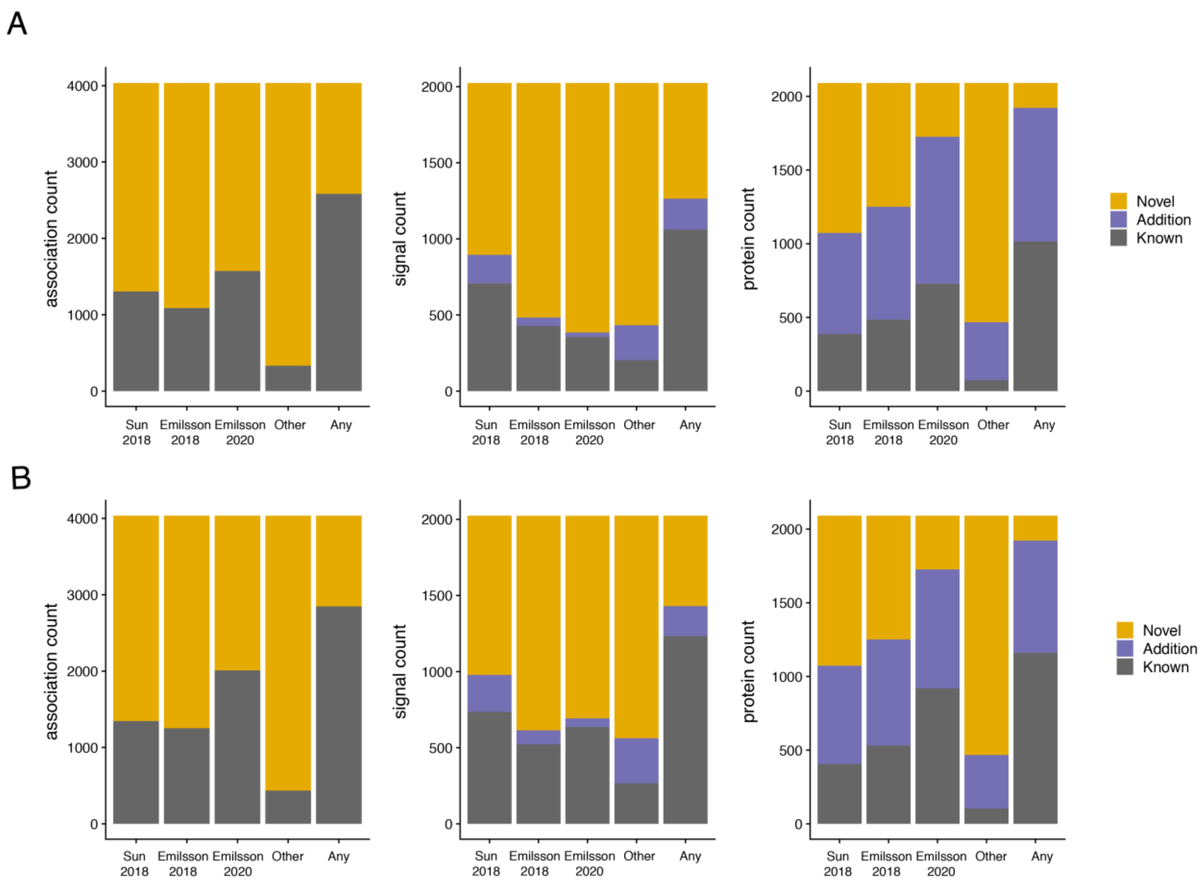


**Supplementary Fig. 5** Comparison of independent study-wide significant pQTLs identified in the current study (n = 5,368) to those previously reported, using (**A**) the primary LD threshold $r^2 > 0.9$ to define shared signals, and (**B**) a more stringent LD threshold $r^2 > 0.5$ to define shared signals. The barplots show if a matching pQTL association (left panel) has been previously reported, if a genetic signal (middle panel) has been reported to associate with the same (known) or another (addition) protein, and if the same (known) or another (addition) genetic signal has previously been reported for a given protein (right panel). See Supplementary Table 1 for exact numbers. An overview of the studies used for comparison is shown in Supplementary Data 5.

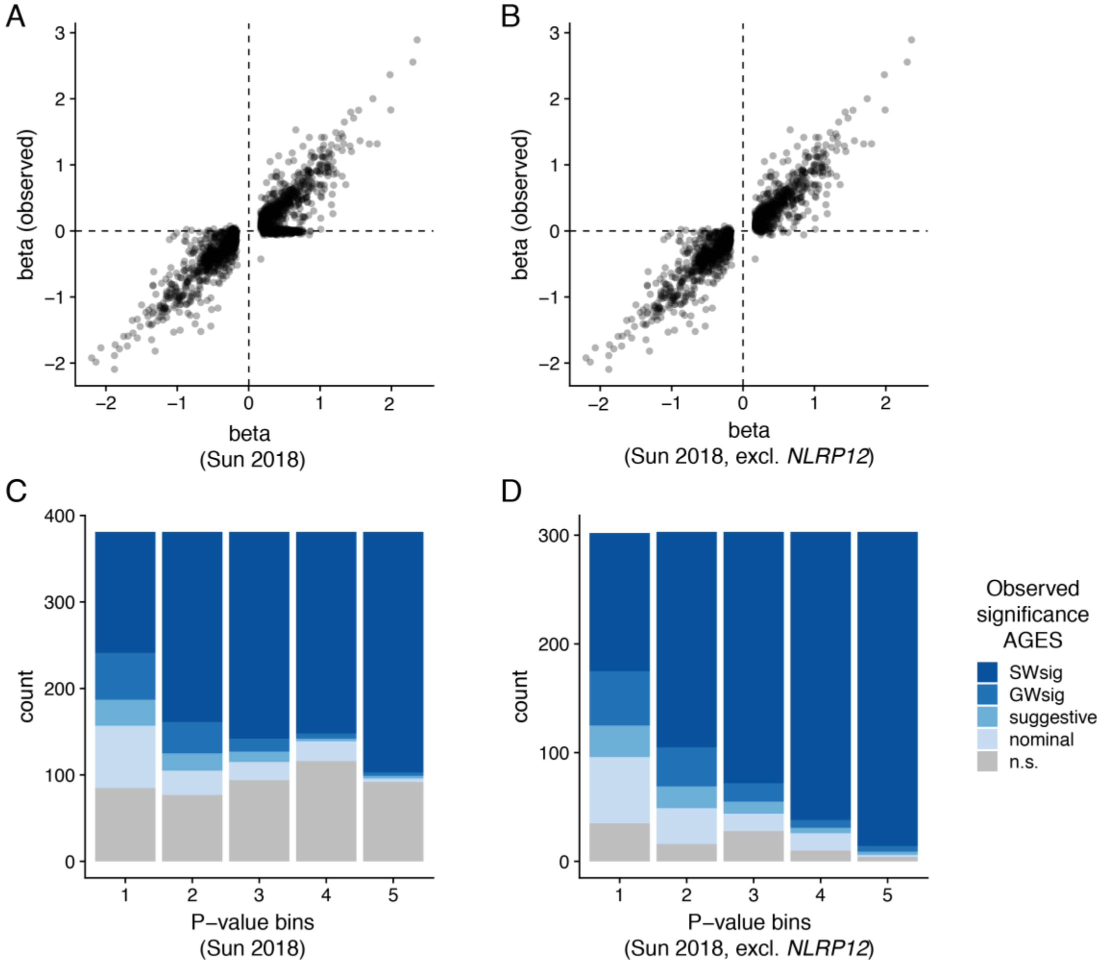


**Supplementary Fig. 6** Replication of previously reported pQTLs from the INTERVAL study[1] in AGES. (**A**-**B**) Comparison of reported pQTL beta values for (**A**) all independent study-wide significant pQTLs reported by Sun et al.[1]. (**B**) all independent study-wide significant pQTLs reported by Sun et al.[1], but excluding the *NLRP12* locus, with matching pQTL beta values observed in the current study (linear regression, n = 5,368). (**C**-**D**) Number of pQTLs colored by observed significance level in the current study and shown by significance bins based on reported pQTL P-values for (**C**) all independent study-wide significant pQTLs reported by Sun et al.[1] and (**D**) all independent study-wide significant pQTLs reported by Sun et al.[1], but excluding the *NLRP12* locus, where bin 5 has the lowest (most significant) reported P-values. SWsig: study-wide significant, $P<5\times10^{-8}/4782$, GWsig: genome-wide significant, $P<5\times10^{-8}$, suggestive: $P<1\times10^{-5}$, nominal: $P<0.05$, n.s.: not significant, $P>0.05$.

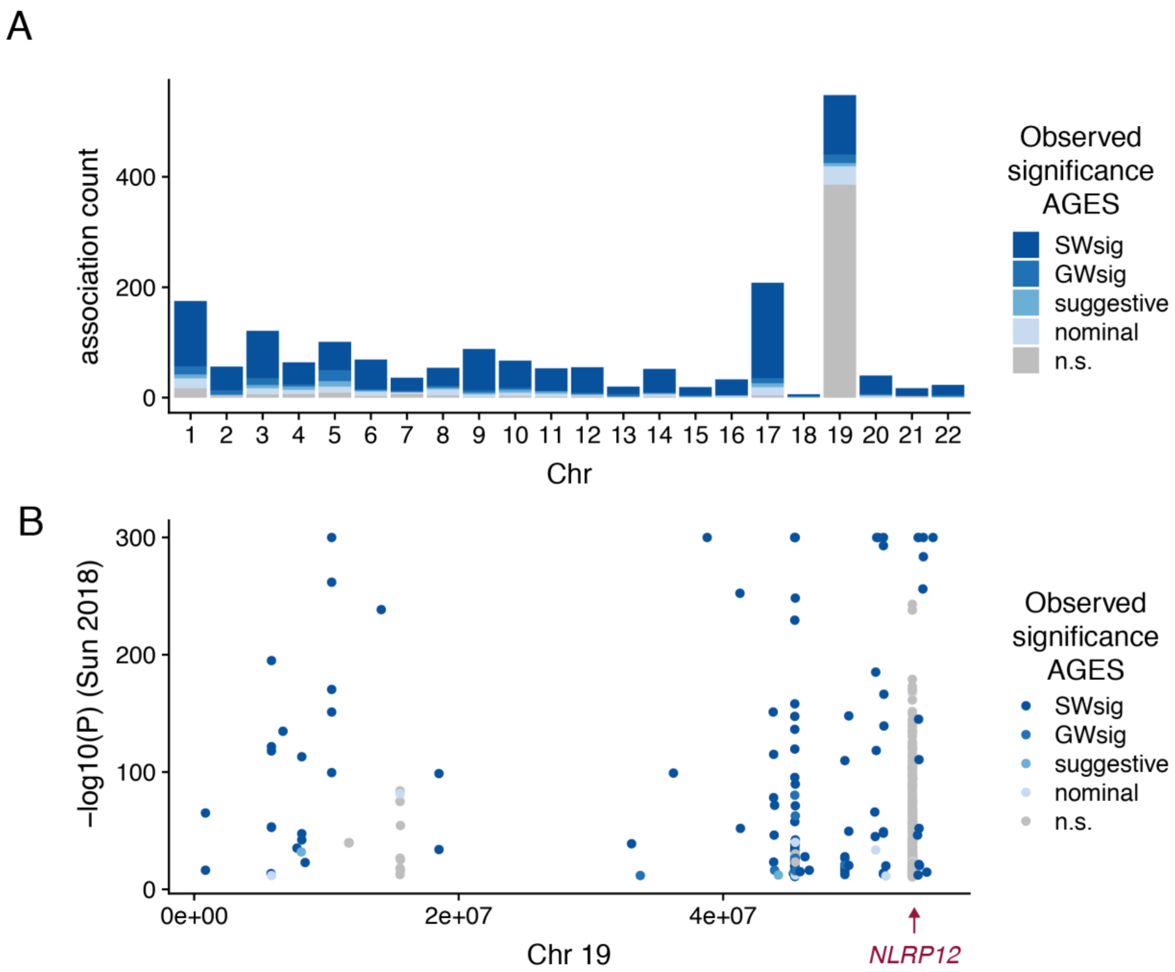


**Supplementary Fig. 7** (**A**) Number of reported independent study-wide significant associations (pQTLs) by Sun et al.[1] from the INTERVAL study by chromosome, colored by observed significance level in AGES (n = 5,368). (**B**) Independent study-wide significant pQTLs reported by Sun et al.[1] on chromosome 19 colored by significance level in AGES (linear regression, n = 5,368). The *NLRP12* locus is highlighted. SWsig: study-wide significant, $P<5\times10^{-8}/4782$, GWsig: genome-wide significant, $P<5\times10^{-8}$, suggestive: $P<1\times10^{-5}$, nominal: $P<0.05$, n.s.: not significant, $P>0.05$.

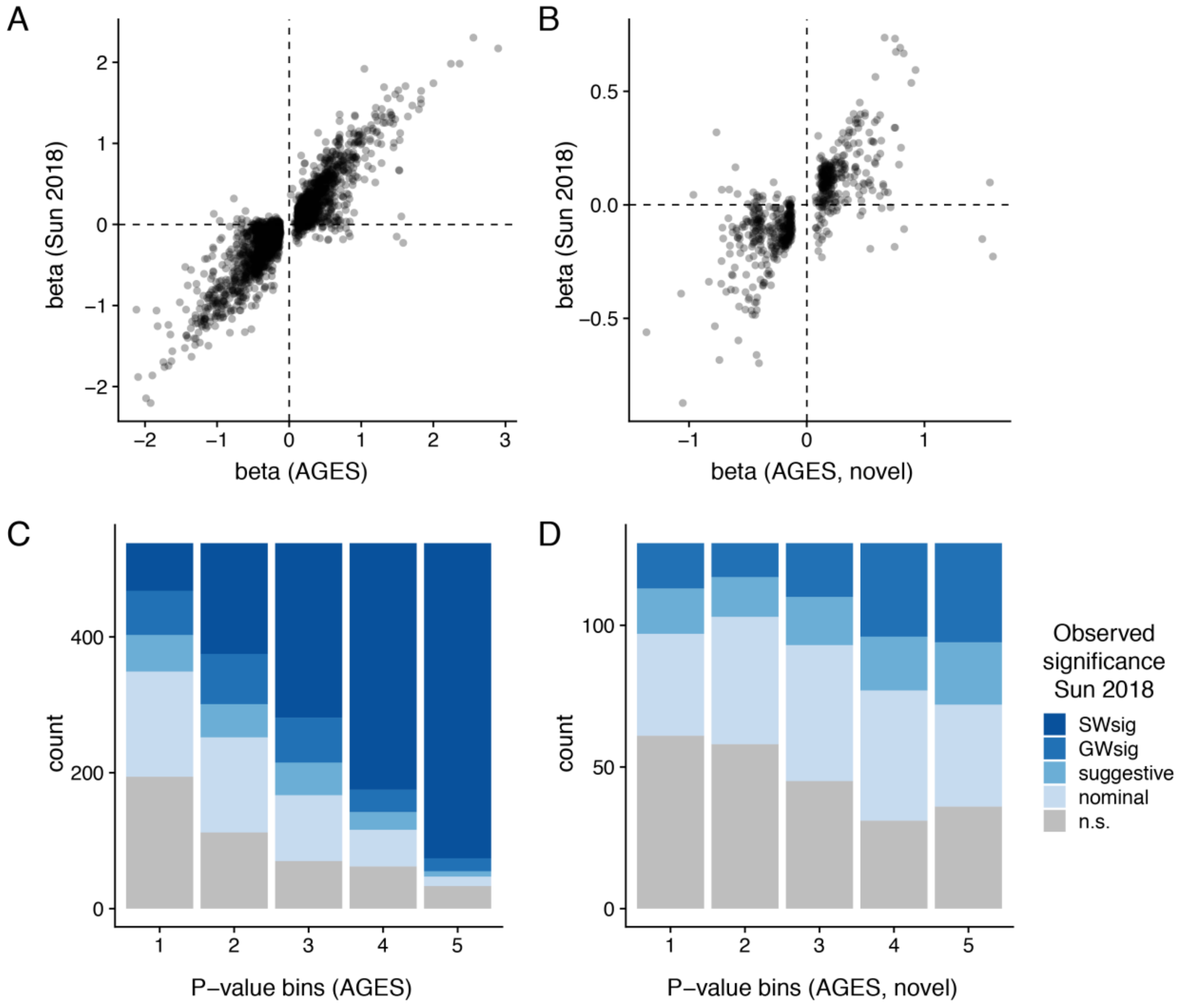


**Supplementary Fig. 8** Replication of pQTLs identified in the current study (linear regression) using summary statistics from the INTERVAL study[1]. (**A**-**B**) Comparison of beta values for (**A**) all independent study-wide significant pQTLs in AGES (conditional and joint analysis, GCTA-COJO) and (**B**) independent study-wide significant pQTLs defined as novel in the current study, with matching pQTL beta values from Sun et al.[1]. (**C**-**D**) Number of pQTLs binned by P-values in the current study, shown for (**C**) all independent study-wide significant pQTLs and (**D**) independent study-wide significant pQTLs defined as novel in the current study, where bin 5 has the lowest (most significant) P-values, colored by significance level in the INTERVAL study[1]. SWsig: study-wide significant, $P<5\times10^{-8}/3{,}283$ (number of SOMAmers in INTERVAL study[1]), GWsig: genome-wide significant, $P<5\times10^{-8}$, suggestive: $P<1\times10^{-5}$, nominal: $P<0.05$, n.s.: not significant, $P>0.05$.

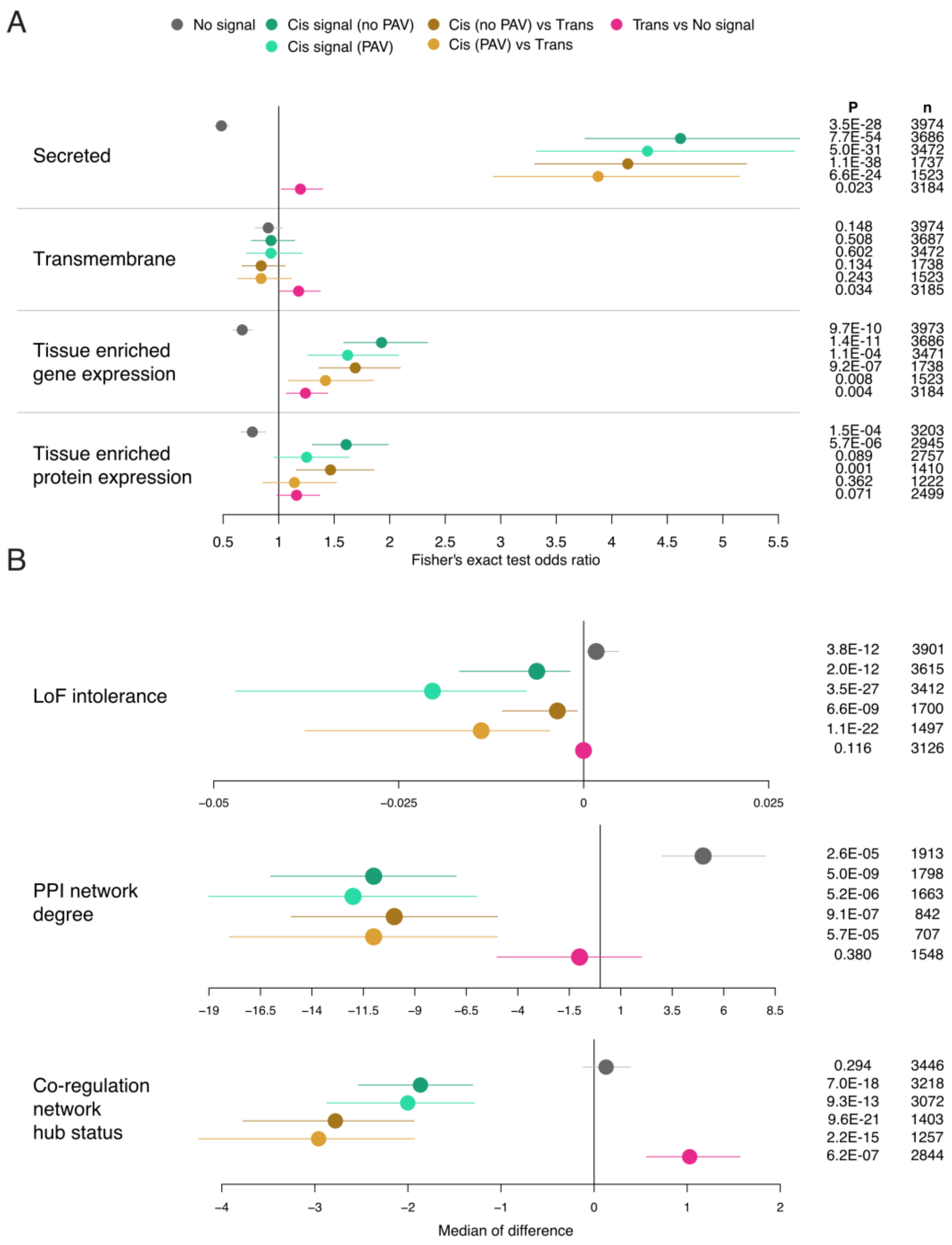


**Supplementary Fig. 9** Enrichment analysis comparing characteristics between proteins classified by types of genetic association signals, where *cis*-signals are stratified by tagging a protein-altering variant (PAV) affecting the corresponding gene for the protein target or not and compared to those without any *cis*-signal. See Methods text for definitions. (**A**) Fisher's exact test (two-sided) for comparing two classifications. Odds ratio estimates are presented with 95% confidence interval. (**B**) Wilcoxon's rank sum test (two-sided) for comparing classifications with continuous traits. Estimates of the median of the difference between values from the two classes are presented with 95% confidence interval. P-values are shown to the right.

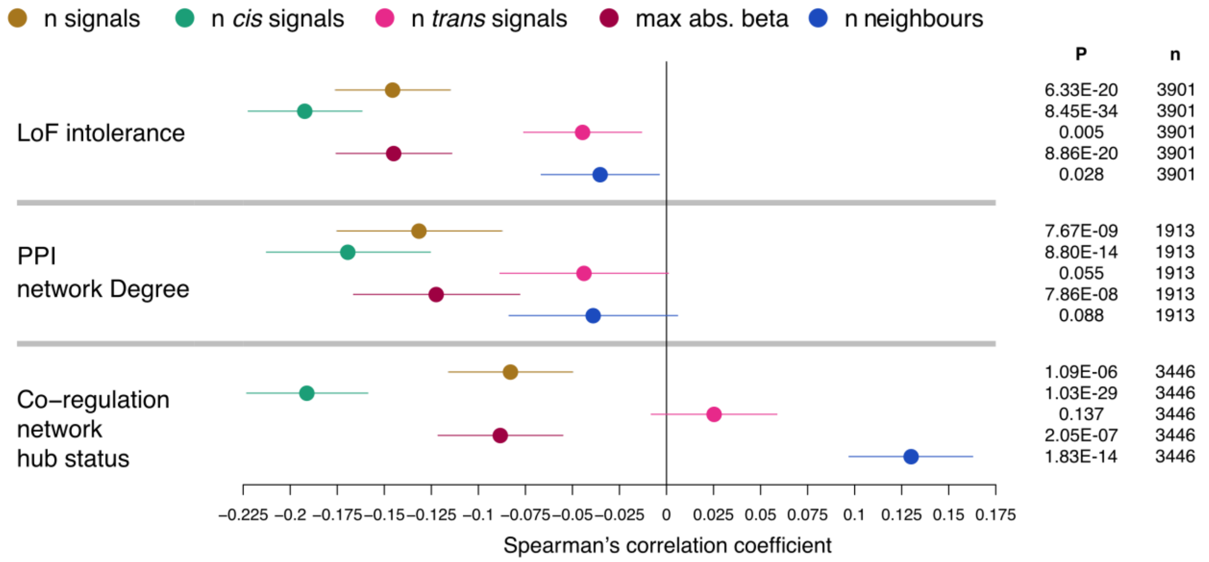


**Supplementary Fig. 10** Comparing genetic association profile characteristics (number of signals in total, cis or trans, largest absolute effect size among study-wide significant independent pQTLs, and number of neighbors defined as the number of proteins that share the same genetic signal) for proteins to metrics of loss-of-function (LoF) intolerance, protein-protein interaction (PPI) network degree and co-regulation network hub status. Spearman's rank correlation coefficient estimates are presented with 95% confidence interval. P-values are calculated via an asymptotic t-distribution approximation (two-sided) and are shown to the right.

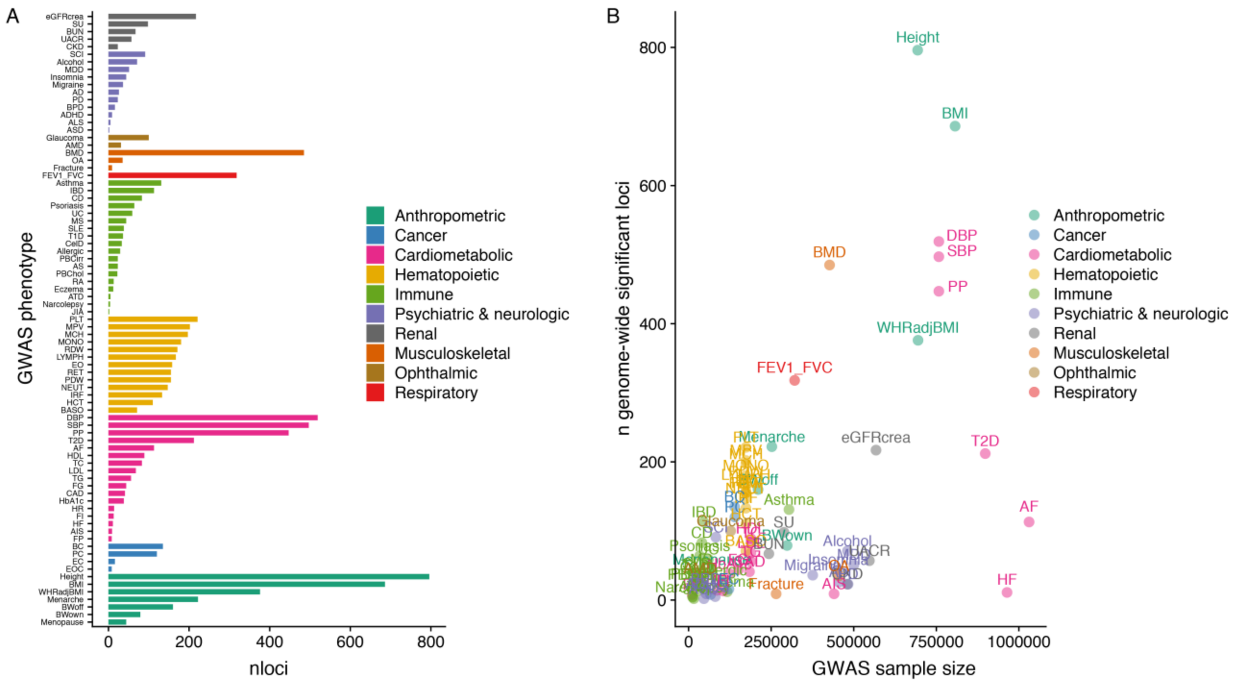


**Supplementary Fig. 11** (**A**) An overview of the number of genome genome-wide significant loci (x-axis) for GWAS of 81 clinical traits and diseases (y-axis) for which summary statistics were included in the colocalization analysis with serum proteins. (**B**) An overview of the GWAS sample sizes (x-axis) and the number of genome-wide significant loci (y-axis) for the 81 clinical traits and diseases for which GWAS summary statistics were included in the colocalization analysis with serum proteins. GWAS for immune traits, in particular, have smaller sample sizes and, as a result, fewer identified loci than other trait categories.

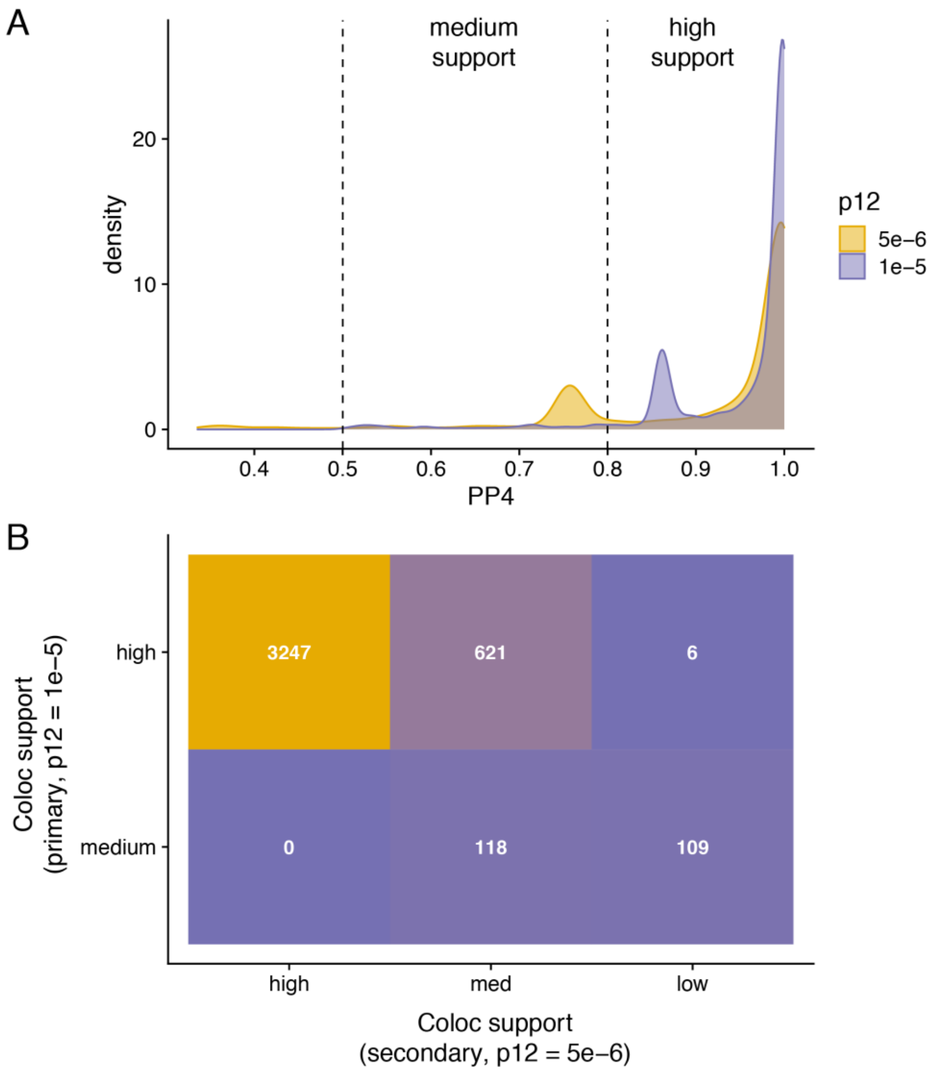


**Supplementary Fig. 12** (**A**) Comparison of posterior probability values for a shared signal (PP4) obtained from coloc in the colocalization analysis for two different priors; the default coloc value of 1e-5 (primary analysis) and a more stringent value of 5e-6 (secondary analysis). The vertical dashed lines indicate the cutoffs used to define medium (PP4>0.5) and high (PP4>0.8) support for colocalization. The more stringent prior shifts some protein-phenotype pairs below the 0.8 cutoff. (**B**) The number of protein-phenotype pairs in each coloc support category using the two different priors in the primary and secondary analysis. Of the 3,874 pairs with high support in the primary analysis, 84% remain so in the secondary analysis with a more stringent prior.

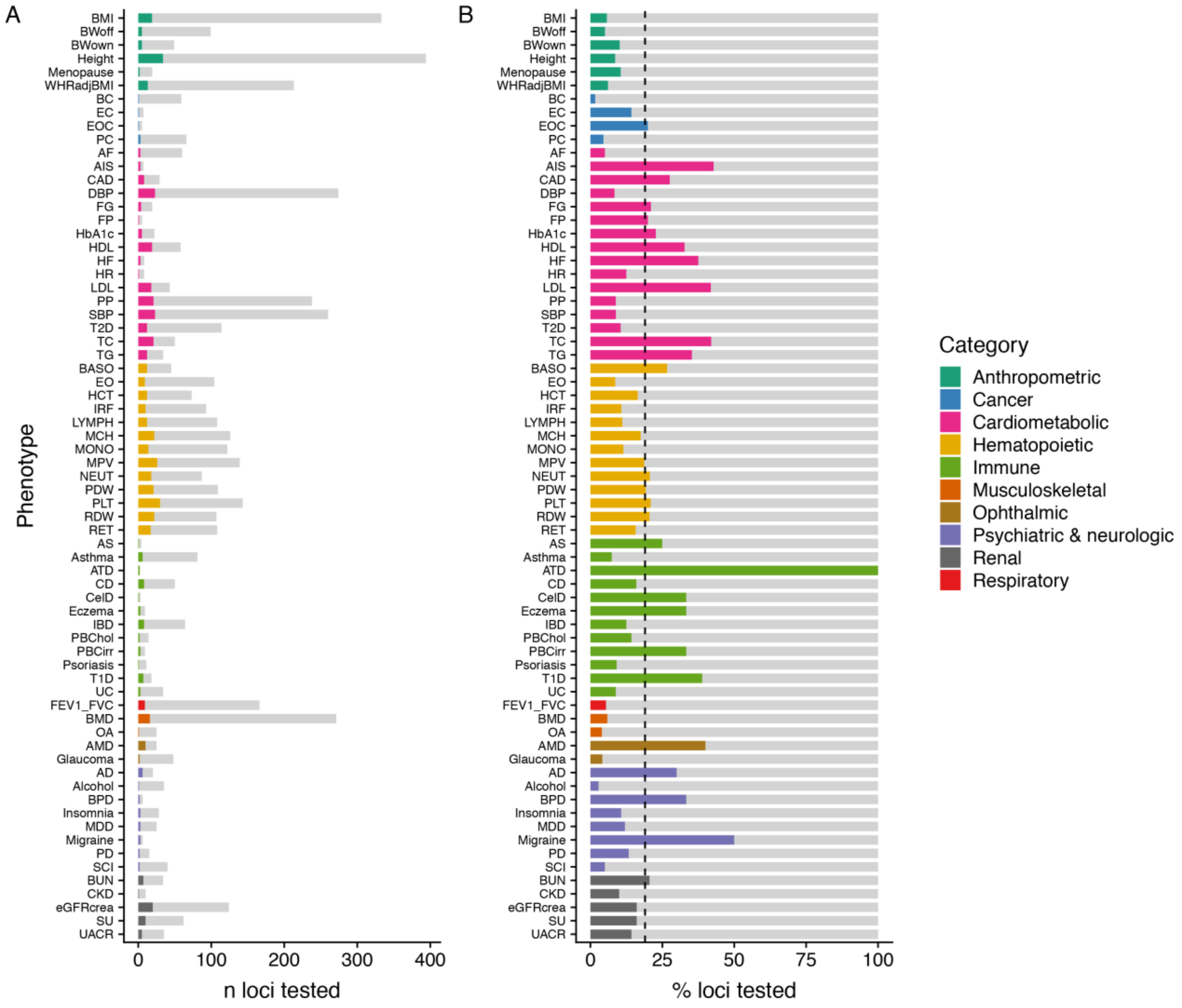


**Supplementary Fig. 13** An overview of the (**A**) number and (**B**) proportion of GWAS loci that colocalize with any serum protein signal in the current study, restricted to the loci that were in the vicinity of a pQTL and thus tested. The dashed line indicates the average for the tested loci, or 19% that colocalize with at least one serum protein (whereas it is 11% when considering all GWAS loci).

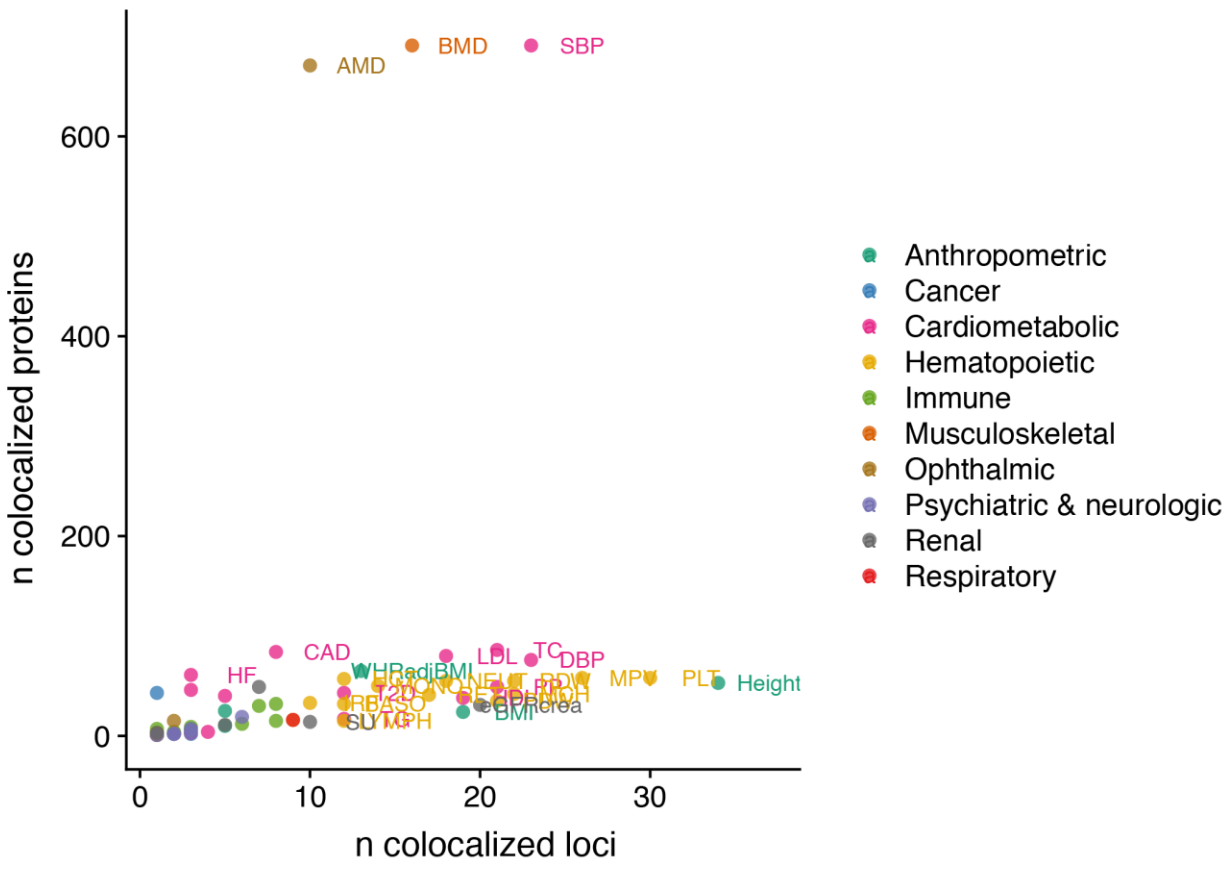


**Supplementary Fig. 14** An overview of the number of colocalized proteins (y-axis) compared to the number of colocalized loci (x-axis) per trait. The three traits (AMD, BMD, SBP) at the top all colocalize with the *VTN* locus, which regulates 597 proteins.

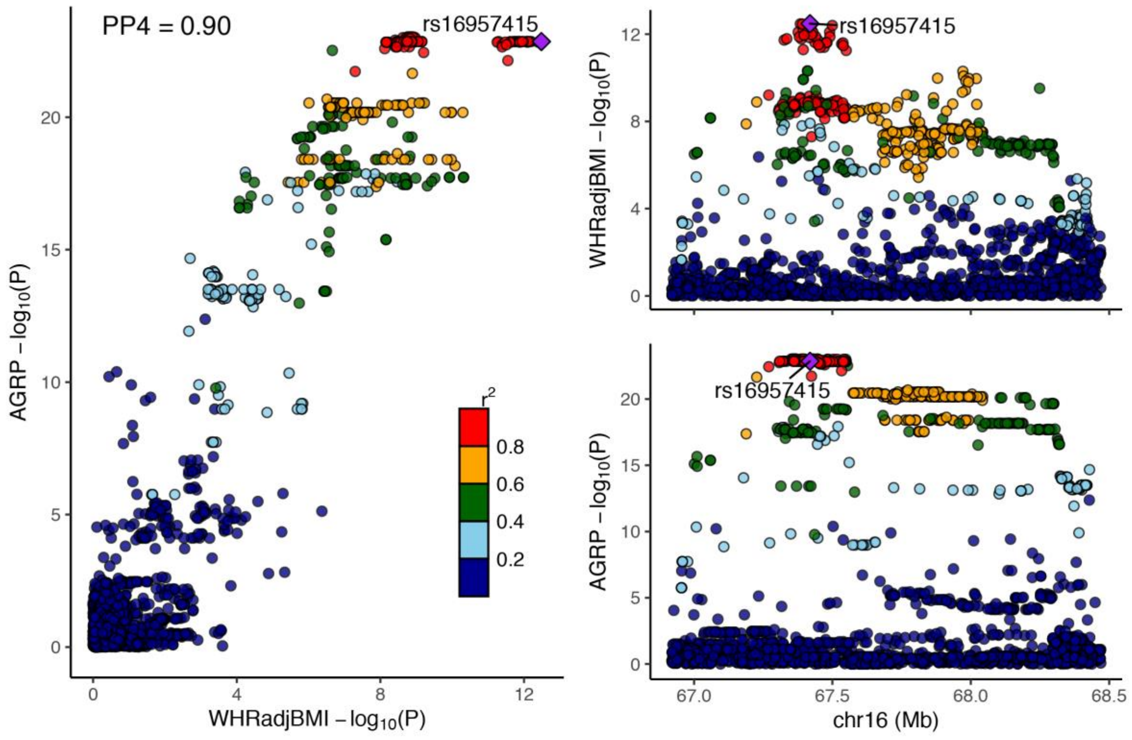


**Supplementary Fig. 15** Colocalization of genetic associations (linear regression) for serum levels of the AGRP protein and waist-to-hip ratio adjusted for BMI (WHRadjBMI) on chromosome 16.

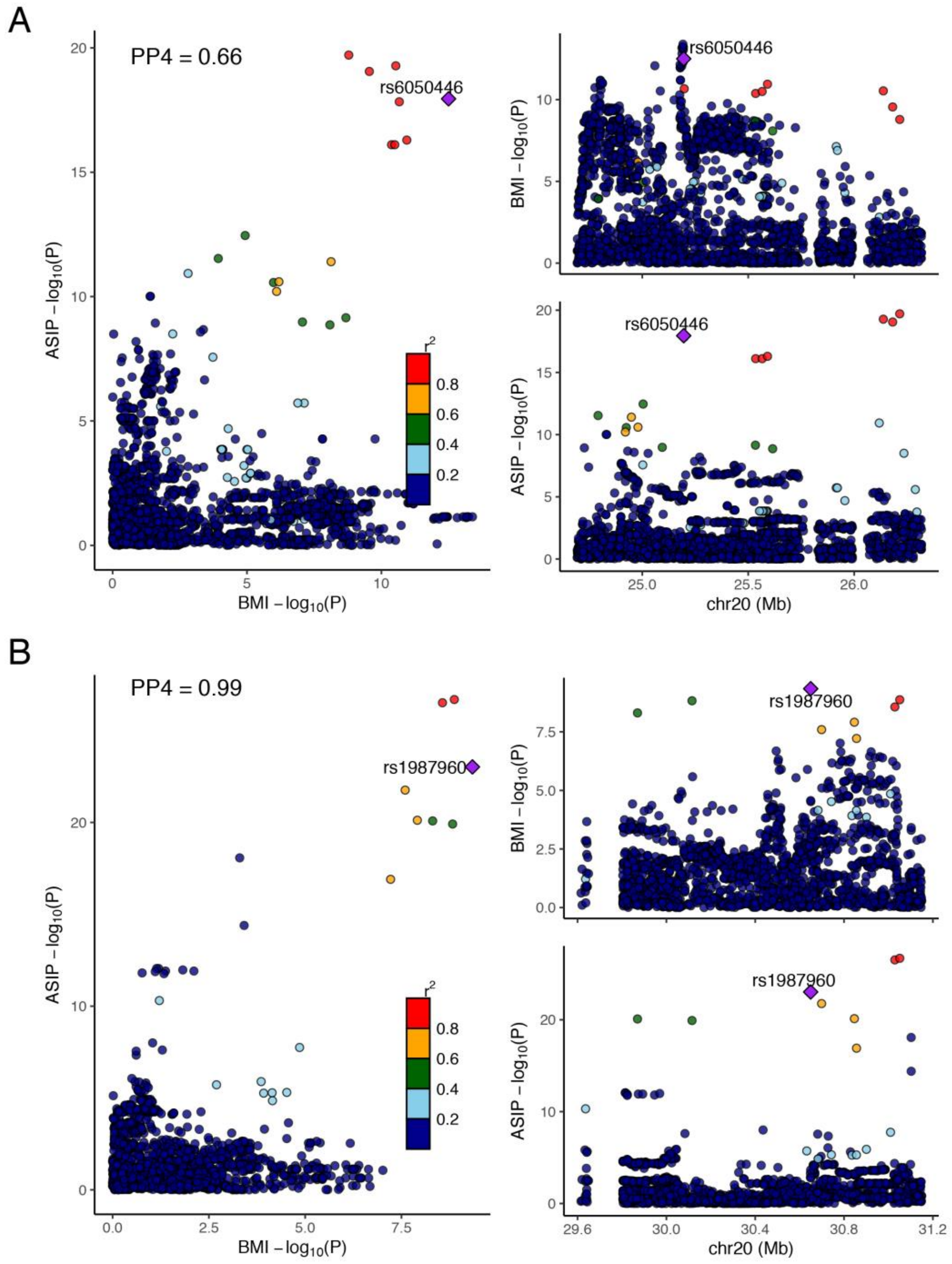


**Supplementary Fig. 16** Colocalization of two genetic associations (linear regression) for serum levels of the ASIP protein and BMI on chromosome 20 at around (**A**) 25.5Mb and (**B**) 30.4Mb.

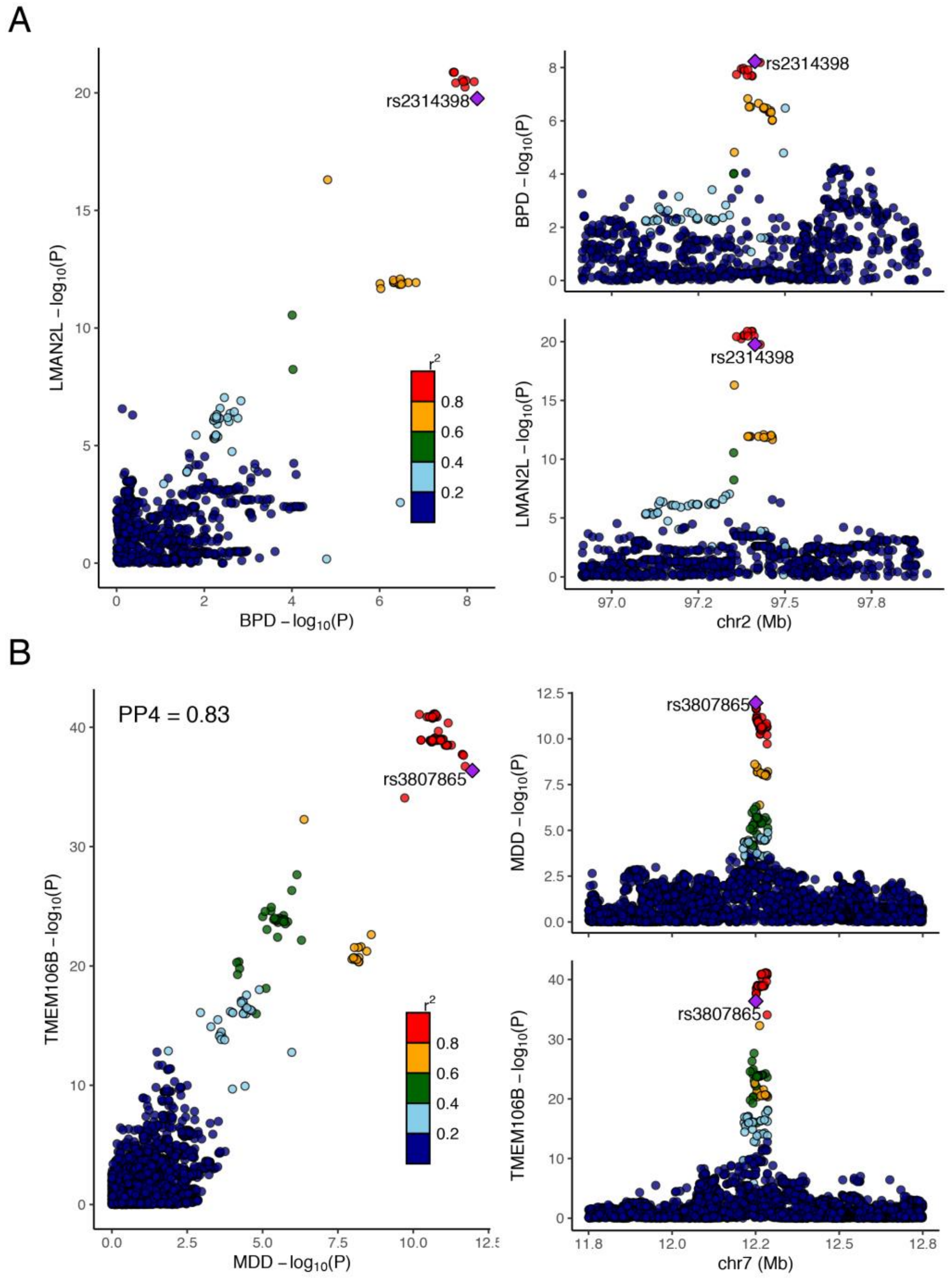


**Supplementary Fig. 17** Colocalization between genetic associations (linear regression) for (**A**) serum levels of the LMAN2L protein and bipolar disorder (BPD) on chromosome 2 and (**B**) serum levels of the TMEM106B protein and major depressive disorder (MDD) on chromosome 7.

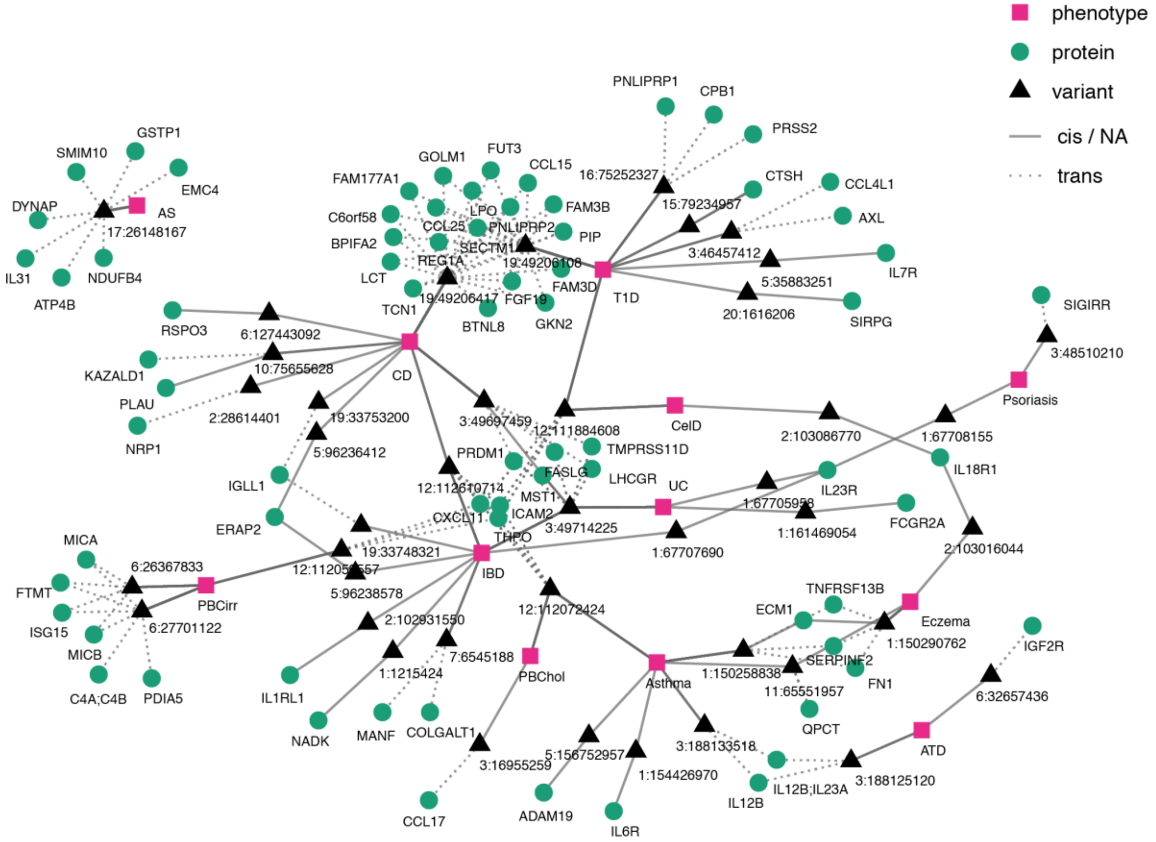


**Supplementary Fig. 18** Immune disease colocalization network, illustrating how diseases (pink squares) are linked through the overlap of colocalized proteins (green circles). Diseases and proteins are linked through lead variants (black triangles) in the loci where genetic signals colocalize, where the pQTL may be in *cis* (solid line) or *trans* (dotted line).

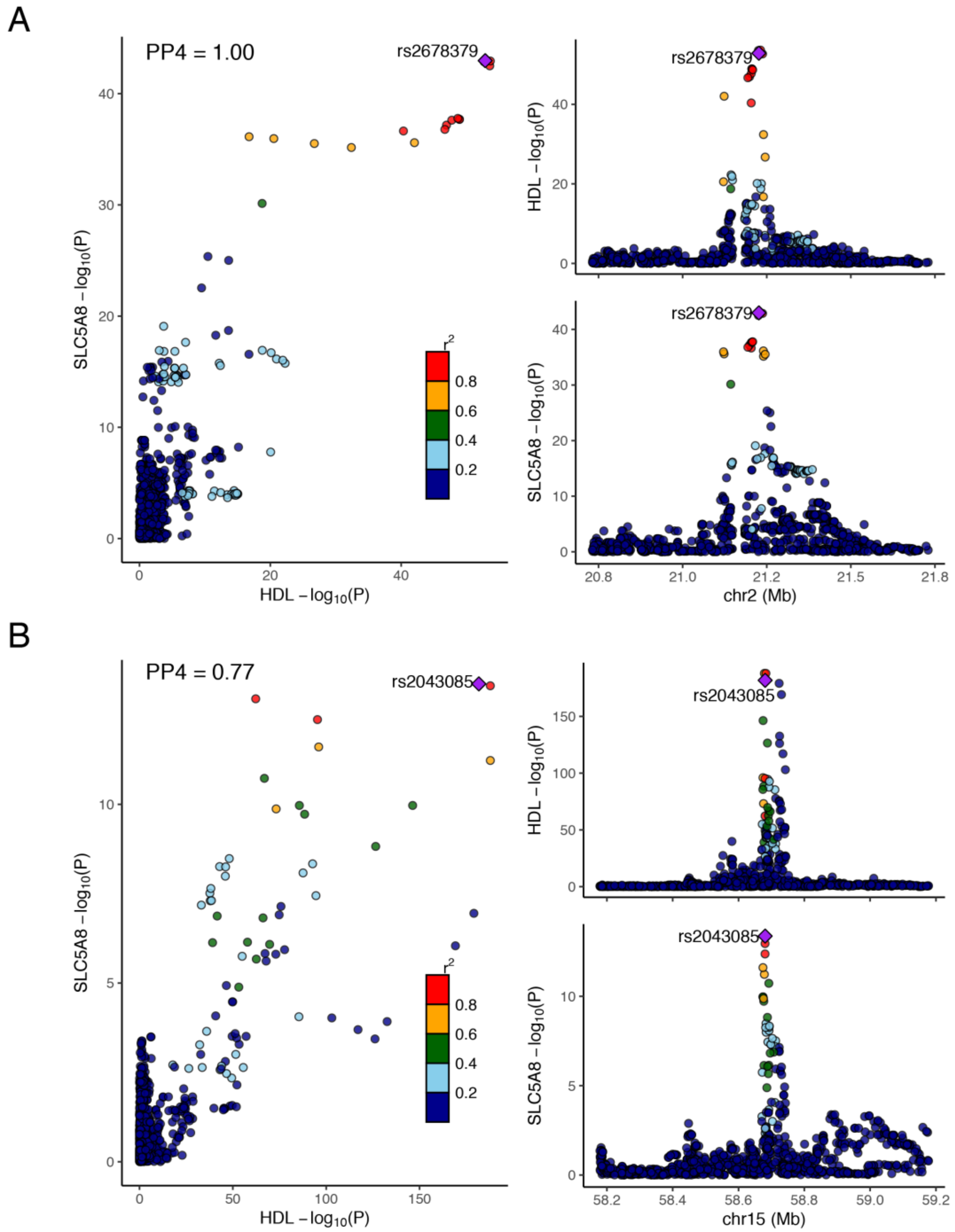


**Supplementary Fig. 19** Colocalization between genetic associations (linear regression) for serum levels of the SLC58A protein and HDL cholesterol on (**A**) chromosome 2 and (**B**) chromosome 15.

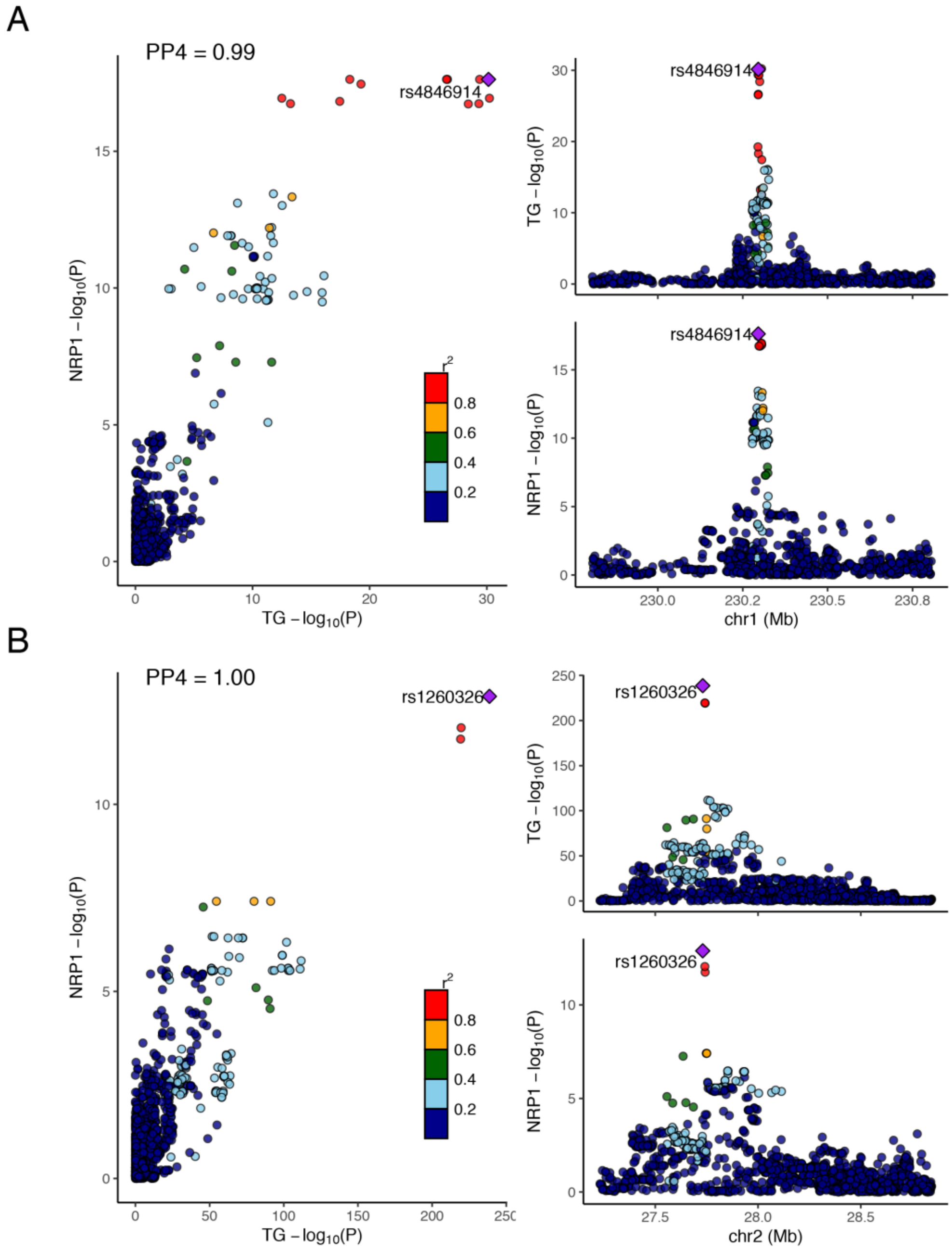


**Supplementary Fig. 20** Colocalization between genetic associations (linear regression) for serum levels of the NRP1 protein and triglycerides (TG) on (**A**) chromosome 1 and (**B**) chromosome 2.

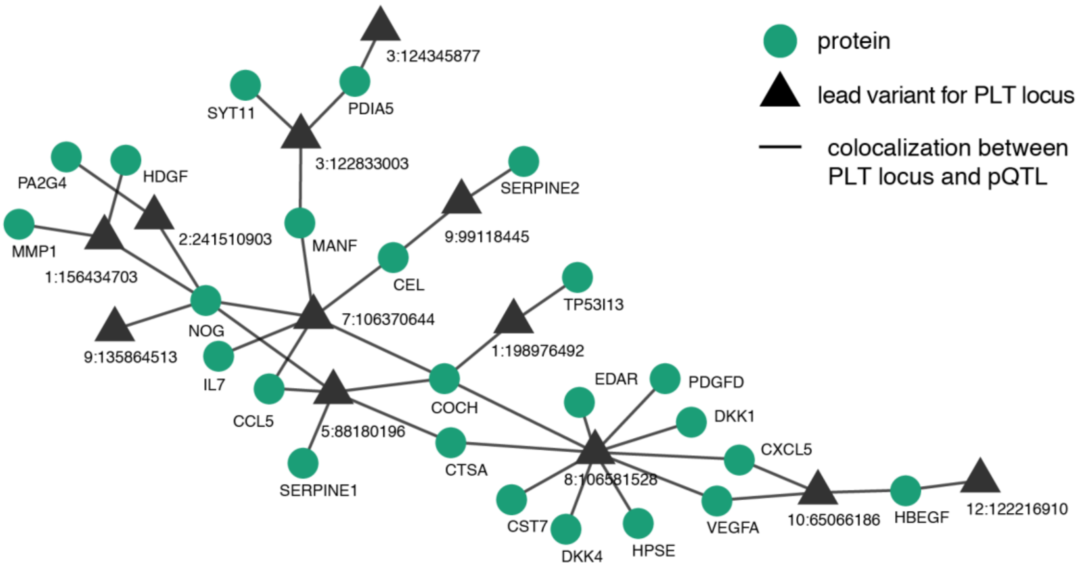


**Supplementary Fig. 21** Platelet colocalization network, illustrating how many of the same proteins (green circles) colocalize with GWAS signals for platelet count (PLT) in multiple loci, represented by lead variants (black triangles). For example, pQTLs for NOG and COCH colocalize with GWAS signals for platelet counts in five and four loci, respectively.

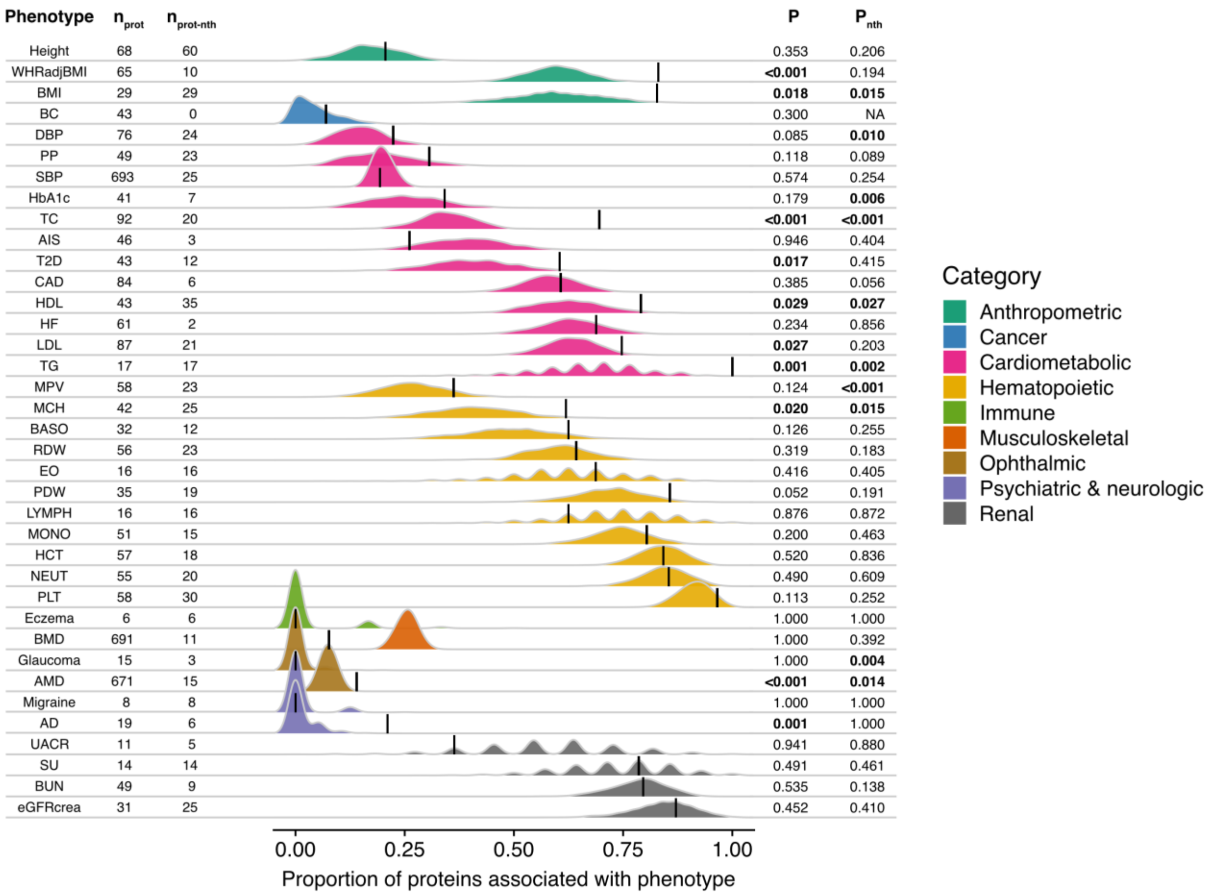


**Supplementary Fig. 22** Ridgeline plot illustrating for each GWAS phenotype the proportion of colocalized proteins including study-wide significant ($P<1.046\times10^{-11}$) pQTLs (linear regression, n = 5,368) that were significantly associated with the same trait in AGES (linear regression, FDR<0.05, n = 5,457) (black lines) compared to 1000 randomly sampled sets of proteins of the same size (density curves). The number of colocalized proteins for each trait are provided on the left-hand side, along with the number of proteins remaining after the removal of proteins originating from loci with 5 or more colocalized proteins from the analysis, annotated as “no trans-hotspots” (nth). Empirical P-values for significant enrichment of protein-phenotype associations are provided to the right. Empirical P-values < 0.05 are highlighted in bold.

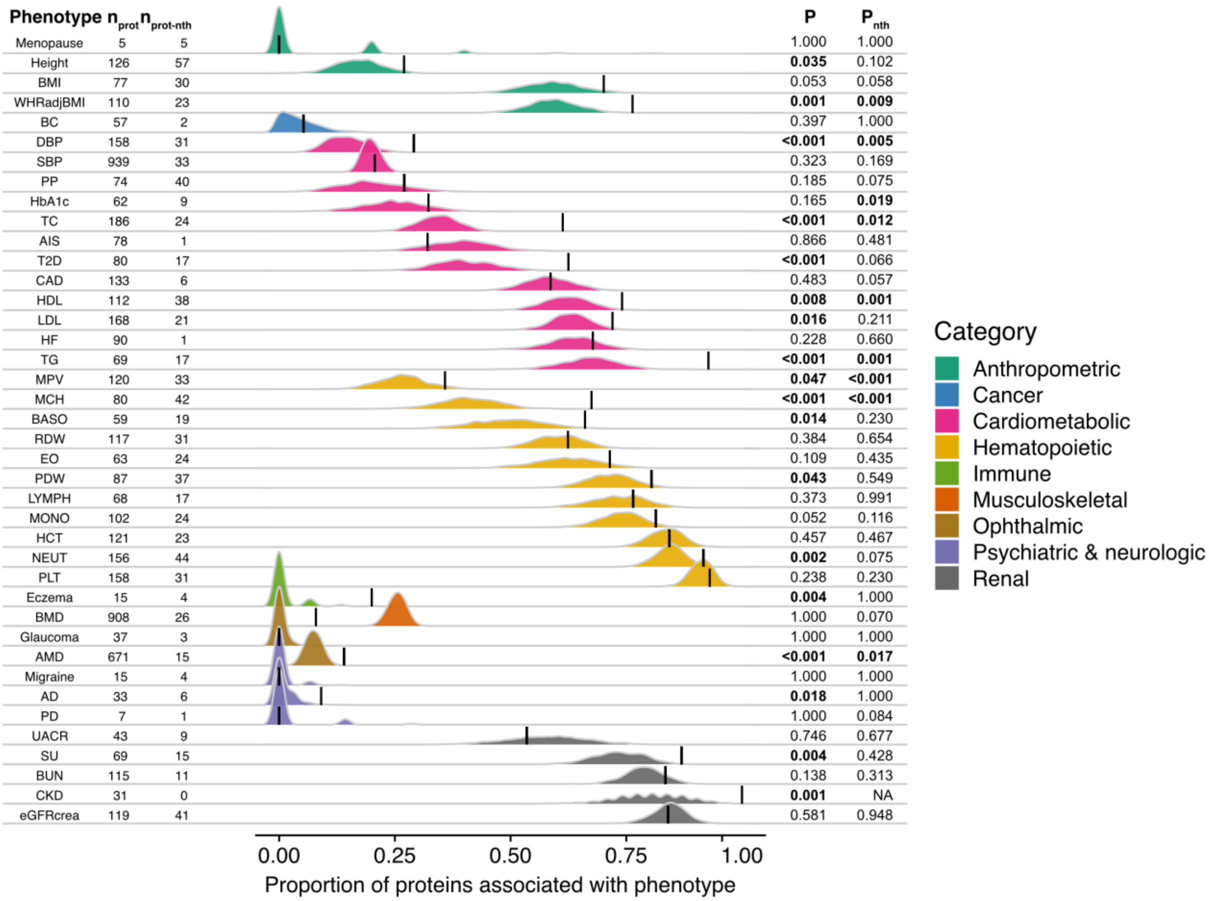


**Supplementary Fig. 23** Ridgeline plot illustrating for each GWAS phenotype the proportion of colocalized proteins including all genome-wide significant ($P<5\times10^{-8}$) pQTLs (linear regression, n = 5,368) that were significantly associated with the same trait in AGES (linear regression, FDR<0.05, n = 5,457) (black lines) compared to 1000 randomly sampled sets of proteins of the same size (density curves). The number of colocalized proteins for each trait are provided on the left-hand side, along with the number of proteins remaining after the removal of proteins originating from loci with 5 or more colocalized proteins from the analysis, annotated as “no trans-hotspots” (nth). Empirical P-values for significant enrichment of protein-phenotype associations are provided to the right. Empirical P-values < 0.05 are highlighted in bold.

## Supplementary References


1. Sun, B. B. *et al.* Genomic atlas of the human plasma proteome. *Nature* **558,** 73–79 (2018).
2. Emilsson, V. *et al.* Co-regulatory networks of human serum proteins link genetics to disease. *Science* **361,** 769–773 (2018).
3. Emilsson, V. *et al.* Coding and regulatory variants affect serum protein levels and common disease. *bioRxiv* 2020.05.06.080440 (2020). doi:10.1101/2020.05.06.080440
4. Raffield, L. M. *et al.* Comparison of Proteomic Assessment Methods in Multiple Cohort Studies. *Proteomics* **20,** (2020).
5. Canela-Xandri, O., Rawlik, K. & Tenesa, A. An atlas of genetic associations in UK Biobank. *Nat Genet* **50,** 1593–1599 (2018).
6. Klarić, L. *et al.* Glycosylation of immunoglobulin G is regulated by a large network of genes pleiotropic with inflammatory diseases. *Sci Adv* **6,** 24 (2020).
7. Gudelj, I., Lauc, G. & Pezer, M. Immunoglobulin G glycosylation in aging and diseases. *Cell Immunol* **333,** 65–79 (2018).
8. Shen, X. *et al.* Multivariate discovery and replication of five novel loci associated with Immunoglobulin G N-glycosylation. *Nat Commun* **8,** 1–10 (2017).
9. Fritsche, L. G. *et al.* A large genome-wide association study of age-related macular degeneration highlights contributions of rare and common variants. *Nat Genet* **48,** 134–143 (2016).
