# Supplementary material for: A genome-wide association study of serum proteins reveals shared loci with common diseases
Source: Nat Commun. 2022 Jan 25;13:480. doi: 10.1038/s41467-021-27850-z (PMC8789779; doi:10.1038/s41467-021-27850-z)
Supplement: Supplementary file 2 — Description of Additional Supplementary Files [file 41467_2021_27850_MOESM2_ESM.pdf]

Description of Additional Supplementary Files

File Name: Supplementary Data 1
Description: AGES-Reykjavik cohort baseline characteristics for all individuals with protein measurements (n = 5,457 of whom 5,368 had genotype data), including all phenotypes used in the protein-phenotype association analysis.

File Name: Supplementary Data 2
Description: The number of study-wide associations between independent genetic signals and proteins, broken down by cis and trans associations.

File Name: Supplementary Data 3
Description: All independent genetic signals at a study-wide significant threshold and their associations to proteins. The BETA, SE and P columns show the pre-conditional linear regression coefficients (effect size, standard error and P-value), while the appended J signifies the coefficients in the conditional-joint analysis from the GCTA-COJO analysis or the validation analysis (linear regression) using individual level data. The Signal column joins genetic variants across proteins in LD > 0.9 and the Locus identifier column joins genetic variants within 300kb distance. The trans-hotspot column indicates if the given signal associates with >= 10 unique proteins.

File Name: Supplementary Data 4
Description: Overview of the loci defined from the independent signals in Table S3 by combining those within 300kb distance

File Name: Supplementary Data 5
Description: Overview of previously published studies used for estimating the novelty of pQTLs reported in the current study

File Name: Supplementary Data 6
Description: Protein annotations used for enrichment analysis. Annotations related to genetic association profile in AGES are based on study-wide independent signals (conditional and joint analysis, GCTA-COJO) and include number and type of signals (no signal, any cis-signal, only trans-signal), most significant observed P-value, largest observed effect size and number of neighbours, defined as proteins that share a genetic signal. External annotations include tissue-enhanced or enriched expression, cellular localization, loss-of-function intolerance and network degree (see Methods for details).

File Name: Supplementary Data 7
Description: Enrichment analysis results. The var1 column contains the variables defined in this study, while the var2 column contains the variables obtained externally. The test column states which statistical test was used, depending on whether the variables were continuous or binary. All tests are two-sided. The estimate column depends on the test column as follows, odds ratio for Fisher's test, rho correlation for Spearman's test and the median of difference between each pair of values for Wilcox's rank sum test. The 95% confidence interval for this estimate is provided Number of proteins (n) included for each test is provided.

File Name: Supplementary Data 8
Description: An overview of the 81 GWAS summary statistics obtained for systematic comparison with protein associations. The "Matched phenotype in AGES" column indicates which phenotype in AGES was

deemed closest to the reported GWAS phenotype and used for the protein-phenotype association analysis.

File Name: Supplementary Data 9
Description: Overview of colocalization results, stratified by coloc support

File Name: Supplementary Data 10
Description: Colocalization results between proteins and GWAS traits with medium (coloc $0.5<PP.H4<=0.8$) or high (coloc $PP.H4>0.8$) support. For each locus, the lead variants for phenotype and protein are listed, defined as the genetic variant within the locus with the smallest P-value from GWAS (linear regression), as well as the best shared variant, defined as the one with the lowest summed P-value from its associations with phenotype and protein. Lead variants here are selected from those with information on both phenotype and protein associations and thus included in the colocalization analysis. We also include a flag that indicates whether the lead pQTL of each locus (or a proxy, LD $r2 > 0.8$) is included in the set of overlapping SNPs for any given trait (included = 1, not included = 0).

File Name: Supplementary Data 11
Description: Overview of conditional colocalization results, highlight the proportion of colocalizing signals that are identified in addition to those in the original colocalization analysis.

File Name: Supplementary Data 12
Description: Conditional colocalization results between proteins and GWAS traits with medium (coloc $0.5<PP.H4<=0.8$) or high (coloc $PP.H4>0.8$) support. For each locus, the lead variants for phenotype and protein are listed, defined as the genetic variant within the locus with the smallest P-value from GWAS (linear regression), as well as the best shared variant, defined as the one with the lowest summed P-value from its associations with phenotype and protein. Lead variants here are selected from those with information on both phenotype and protein associations and thus included in the colocalization analysis.

File Name: Supplementary Data 13
Description: Direct association between proteins and traits in AGES that were found to colocalize in any locus. The associations were tested with a linear or logistic regression adjusted for age and sex. Benjamini-Hochberg adjustment for multiple testing (FDR) was applied per trait. Phenotype matching between reported GWAS and AGES is shown in Table S8.

File Name: Supplementary Data 14
Description: Enrichment analysis of phenotype associations (linear regression) among colocalized proteins per GWAS trait (Table S10) compared to 1000 permutations of randomly sampled protein sets of the same size, from which an empirical P-value was calculated. An empirical P-value <0.001 means that in none of the 1000 randomly sampled protein sets, the proportion of phenotype associated proteins was greater or equal than in the set of colocalized proteins. The analysis was performed with and without loci regulating 5 or more proteins.

File Name: Supplementary Data 15
Description: Enrichment analysis of phenotype associations (linear regression) among colocalized proteins per trait and locus, compared to 1000 permutations of randomly sampled protein sets of the same size, from which an empirical P-value was calculated. An empirical P-value <0.001 means that in none of the 1000 randomly sampled protein sets, the proportion of phenotype associated proteins was

greater or equal than in the set of colocalized proteins. The analysis was restricted to loci regulating 5 or more proteins.

File Name: Supplementary Data 16
Description: GWAS catalogue accession IDs for each summary statistics dataset based on unique pQTL/SOMAmer
