# Supplementary material for: A genome-wide association study of serum proteins reveals shared loci with common diseases
Source: Nat Commun. 2022 Jan 25;13:480. doi: 10.1038/s41467-021-27850-z (PMC8789779; doi:10.1038/s41467-021-27850-z)
Supplement: Supplementary file 19 — Reporting Summary [file 41467_2021_27850_MOESM19_ESM.pdf]

nature research

Corresponding author(s): Vilmundur Gudnason

Last updated by author(s): November 18, 2021

Reporting Summary

Nature Research wishes to improve the reproducibility of the work that we publish. This form provides structure for consistency and transparency in reporting. For further information on Nature Research policies, see our Editorial Policies and the Editorial Policy Checklist.

Statistics

For all statistical analyses, confirm that the following items are present in the figure legend, table legend, main text, or Methods section.

n/a	Confirmed	
☐	☒	The exact sample size (*n*) for each experimental group/condition, given as a discrete number and unit of measurement
☐	☒	A statement on whether measurements were taken from distinct samples or whether the same sample was measured repeatedly
☐	☒	The statistical test(s) used AND whether they are one- or two-sided<br>*Only common tests should be described solely by name; describe more complex techniques in the Methods section.*
☐	☒	A description of all covariates tested
☐	☒	A description of any assumptions or corrections, such as tests of normality and adjustment for multiple comparisons
☐	☒	A full description of the statistical parameters including central tendency (e.g. means) or other basic estimates (e.g. regression coefficient) AND variation (e.g. standard deviation) or associated estimates of uncertainty (e.g. confidence intervals)
☐	☒	For null hypothesis testing, the test statistic (e.g. *F*, *t*, *r*) with confidence intervals, effect sizes, degrees of freedom and *P* value noted<br>*Give P values as exact values whenever suitable.*
☐	☒	For Bayesian analysis, information on the choice of priors and Markov chain Monte Carlo settings
☒	☐	For hierarchical and complex designs, identification of the appropriate level for tests and full reporting of outcomes
☐	☒	Estimates of effect sizes (e.g. Cohen's *d*, Pearson's *r*), indicating how they were calculated

Our web collection on statistics for biologists contains articles on many of the points above.

Software and code

Policy information about availability of computer code

Data collection	No software was used for data collection.
Data analysis	R (3.5.1, 4.0.1), RStudio (1.1.456), Minimac3, GCTA (1.92.4beta2), bcftools (1.9), PLINK (1.9, 2.0), GCTA (1.92.4beta2), Ensembl Variant Effect Predictor (104), Circos (0.69), gProfiler, coloc (3.2.1, 4.0.4), LocusComparer (1.0.0)

For manuscripts utilizing custom algorithms or software that are central to the research but not yet described in published literature, software must be made available to editors and reviewers. We strongly encourage code deposition in a community repository (e.g. GitHub). See the Nature Research guidelines for submitting code & software for further information.

Data

Policy information about availability of data

All manuscripts must include a data availability statement. This statement should provide the following information, where applicable:

- Accession codes, unique identifiers, or web links for publicly available datasets
- A list of figures that have associated raw data
- A description of any restrictions on data availability

The custom-design Novartis SOMAscan is available through a collaboration agreement with the Novartis Institutes for BioMedical Research (lori.jennings@novartis.com). Data from the AGES Reykjavik study are available through collaboration (AGES_data_request@hjarta.is) under a data usage agreement with the IHA. All access to data is controlled via the use of a subject-signed informed consent authorization. The time it takes to respond to requests varies depending on their nature and circumstances of the request, but it will not exceed 14 working days. The protein GWAS summary statistics data in this study have been deposited in the GWAS catalog database under the accession ID GCP000257. Supplementary Data 16 contains unique accession IDs for each summary statistics data set based on unique SOMAmers. SNP correlations at protein-associated loci from the AGES cohort are available from zenodo.org (https://doi.org/10.5281/zenodo.5711426). All other data supporting the conclusions of the paper are presented in the main text and freely available as a supplement to

this manuscript (Supplementary Information and Supplementary Data).

Field-specific reporting

Please select the one below that is the best fit for your research. If you are not sure, read the appropriate sections before making your selection.

- [x] Life sciences
- [ ] Behavioural & social sciences
- [ ] Ecological, evolutionary & environmental sciences

For a reference copy of the document with all sections, see nature.com/documents/nr-reporting-summary-flat.pdf

Life sciences study design

All studies must disclose on these points even when the disclosure is negative.

Sample size	The study included all 5457 individuals from the single-center prospective population-based AGES-Reykjavik study for whom protein measurements, of whom 5368 had genotype data available for the GWAS.
Data exclusions	No individuals were excluded.
Replication	We present a two-way pQTL replication analysis comparing current study results to the INTERVAL study results (Sun et al, Nature 2018). Here, 75.6% of pQTLs reported by Sun et al replicated in AGES, i.e. were directionally consistent and nominally significant ($P < 0.05$), with replication increasing to 93.9% when the platform-specific NLRP12 locus was excluded. For the other direction, 83% of the pQTLs identified in AGES and with information in the INTERVAL study were replicated.
Randomization	The participants in this study were not randomized into experimental groups. More to the point, AGES-RS is a population-based study of survivors from the 40-year-long prospective Reykjavik study (random sample of 30,795 individuals), an epidemiologic study aimed at understanding aging in the context of gene/environment interactions by focusing on four biologic systems: vascular, neurocognitive (including sensory), musculoskeletal, and body composition/metabolism.
Blinding	Blinding was not relevant as this study did not compare experimental groups.

Reporting for specific materials, systems and methods

We require information from authors about some types of materials, experimental systems and methods used in many studies. Here, indicate whether each material, system or method listed is relevant to your study. If you are not sure if a list item applies to your research, read the appropriate section before selecting a response.

Materials & experimental systems

n/a	Involved in the study	
x		Antibodies
x		Eukaryotic cell lines
x		Palaeontology and archaeology
x		Animals and other organisms
	x	Human research participants
x		Clinical data
x		Dual use research of concern

Methods

n/a	Involved in the study	
x		ChIP-seq
x		Flow cytometry
x		MRI-based neuroimaging

Human research participants

Policy information about studies involving human research participants

Population characteristics	The study included 5457 Icelandic individuals, i.e. 2330 males (mean age 76.69 years) and 3127 females (mean age 76.52 years), from the population based AGES-Reykjavik study. All AGES study cohort members are European Caucasians.
Recruitment	The Reykjavik Study originally comprised a random sample of 30,795 men and women born in 1907–1935 and living in Reykjavik in 1967. In 2002, the surviving individuals were invited to participate in the AGES-Reykjavík study, which concluded in February 2006 with a total sample size of 5,764 survivors of the Reykjavik Study cohort. See Harris et al., Am J Epidemiol 2007;165:1076–1087.
Ethics oversight	The AGES-Reykjavik study was approved by the National Bioethics Committee in Iceland (approval number VSN-00-063), the National Institute on Aging Intramural Institutional Review Board (US), and the Data Protection Authority in Iceland.

Note that full information on the approval of the study protocol must also be provided in the manuscript.
